# Untargeted Analysis for Mycosporines and Mycosporine-Like Amino Acids by Hydrophilic Interaction Liquid Chromatography (HILIC)—Electrospray Orbitrap MS^2^/MS^3^

**DOI:** 10.3390/antiox9121185

**Published:** 2020-11-26

**Authors:** Maroussia Parailloux, Simon Godin, Susana C. M. Fernandes, Ryszard Lobinski

**Affiliations:** IPREM, UMR 5254, E2S UPPA, CNRS, Universite de Pau et des Pays de l’Adour, 64000 Pau, France; simon.godin@univ-pau.fr (S.G.); susana.fernandes@univ-pau.fr (S.C.M.F.); ryszard.lobinski@univ-pau.fr (R.L.)

**Keywords:** algae, mycosporines, mycosporine-like amino acids, high resolution mass spectrometry, fragment ion search, untargeted screening

## Abstract

Mycosporines and mycosporine-like amino acids have been described as natural sunscreens and antioxidant compounds presenting a great potential for health and cosmetic applications. Herein, an untargeted screening approach for mycosporines and mycosporine-like amino acids (MAAs) was developed by the coupling of zwitterionic hydrophilic interaction liquid chromatography (HILIC) with multistage electrospray mass spectrometry MS^2^/MS^3^ using an Orbitrap analyzer and fragment ion search (FISh). This method was applied to study the mycosporine and MAA contents of five algae extracted using a 50% methanol solution and sonication. Candidate-MAAs were detected by mining eight characteristic fragment ions in their HILIC data-dependent MS^2^ mass spectrum. Their exact masses were measured with 3 ppm mass accuracy and their structures were elucidated on the basis of the MS^3^/MS^4^ mass spectra. The method developed was validated with a targeted analysis using an extract of *Gymnogongrus devoniensis* which confirmed the detection of 14 MAAs reported in the literature. In addition, 23 previously unreported MAAs were detected and the structures could be assigned for seven of them. The developed method was applied to the analysis of four algae: *Gelidium sesquipedale*, *Halopithys incurva*, *Porphyra rosengurtii* and *Cystoseira tamariscifolia* allowing the detection of MAAs, including some reported here for the first time.

## 1. Introduction

Algae inhabiting the intertidal zones with an extensive sun-exposure cope with UV-induced oxidative stress by the synthesis of potent photoprotectants including pigments, phenolic compounds and mycosporine-like amino acids (MAAs) [1]. These water-soluble and small-sized compounds (<500 Da) exhibit strong UV-absorbing properties (molar absorption coefficients ε reaching 50,000 l.M^−1^.cm^−1^) [2]. In the cells, they act mainly as scavengers of radical oxygen species (ROS), prevent lipid oxidation and quench UVB (280–315 nm) and UVA (315–360 nm) radiations without generating oxidative photoproducts [3,4]. These characteristics make MAAs attractive natural products to replace mineral and synthetic UV-filters (sunscreens) in cosmetics and, to be used in UV-protective additives in contact lenses, outdoor materials, textiles, food and drug packaging, and coatings [2,5,6,7,8]. 

MAAs are usually classified in five main families [9] based on their amino-substituent on the C3 as shown in Table 1.

Mycosporines differ from MAAs by the presence of a ketone instead of an imino-group on the C_1_ of their skeleton ring, hence their naming oxo- and imino-MAAs in the literature [9,10]. Please note that the glycine on the C_3_ [10] can be replaced by serine, glutamine or glutamic acid to constitute three atypical MAA subfamilies. In marine micro- and macro-algae, MAAs belong mainly to the palythine and glycine families [9]. MAAs containing sulfate esters such as e.g., mycosporine-taurine or palythine-serine-sulfate, and glycosidic linkages were also identified in cyanobacteria, diatoms, corals and anemones [9,10,11].

The studies of MAAs were originally based on high-performance liquid chromatography (HPLC) using diode array detection (DAD) making use of their high molar absorption coefficient [12,13,14] and the dependence of the absorption wavelength on their amino-cyclohexenimine ring (imino-MAAs, λmax 360 nm) or their amino-cyclohexenone ring (mycosporine or oxo-MAAs, λmax 310 nm) [10]. The identification of the substituent functional groups could be achieved only after chromatographic purification of the individual MAAs followed by their analysis by nuclear magnetic resonance (NMR) and tandem mass spectrometry (ESI-MS^n^) analysis. ^2^H [15] and ^13^C [16] NMR was used for the MAA structure elucidation. Moreover, an ESI-MS^2^ method was developed with hydrogen/deuterium exchange to elucidate unambiguously the MAA structures [17,18].

The isolation and structure elucidation allowed the indexation of over 20 MAAs with their molecular mass, structures, UV-Vis absorption properties and concentrations in more than 500 algal species in the literature and online databases [19]. Reference standards for some of them have been made available which allows their identification on the basis of the retention time using HPLC with diode array (DAD) [14,20,21,22] or quadrupole (Q) ESI-MS detection [10]. Standardless identification requires the on-line detection either by ion-trap MS^2^/MS^3^ or [21] Q—TOF [15,16,23] or high resolution (>100,000) high mass accuracy (<5 ppm) (HRAM) MS. The LC-MS methods used previously and the MAAs identified to date are summarized in Table 2.

The use of HRAM detection has been scarce; the only example is the use of Orbitrap MS in full MS mode to identify five MAAs in two red algae *Hydropuntia cornea* and *Gracilaria longissima* [25]. 

The diversity of the identified MAA structures make plausible the hypothesis that many more still remain to be discovered. The exhaustive information on the MAA diversity is crucial for the prediction of UV-filtering properties of algal extracts and the wider use of algae in cosmetic, biomedical, and industrial fields. It can be obtained by untargeted MS allowing the discovery of unreported MAAs. Hence, the purpose of this research was to develop a HRAM multistage mass spectrometry method and the related data mining strategies for the analysis for MAAs.

## 2. Materials and Methods 

### 2.1. Biological Materials, Chemicals and Reagents 

Purified MAAs, namely shinorine (*Gymnogongrus devoniensis*), palythine (from *Asparragopsis armata*), porphyra-334 (from *Porphyra rosengurtii*), and mycosporine-serinol (from *Lichina pygmaea* lichen), were purchased from the Laboratory of Photobiology of the Central Research Services of the University of Malaga (Spain) and stored at −20 °C. The algae and lichen were collected in 2018 and 2019 in the Andalusian coast (Spain). 

Among the five algal concentrated extracts analyzed, two were provided by the laboratory described above, namely lyophilized algal powders from *Gymnogongrus devoniensis* and *Porphyra rosengurtii*; and the other three were extracted in-house (as described below) from two fresh red algal species (*Gelidium sesquipedales*, *Halopithys incurva*) and one fresh brown algal specie (*Cystoseira tamariscifolia*) collected in November 2018 in the intertidal zone of Les Viviers Basques at Hendaye (France) and stored at −80 °C.

Solutions of purified MAAs were prepared at 5 µg.mL^−1^ in 1.5% methanol and 0.5% acetic acid. Algal concentrated extracts were prepared by extraction with water from stock lyophilized powders of the algae *Gymnogongrus devoniensis* (58 mg.mL^−1^) and *Porphyra rosengurtii* (22 mg.mL^−1^). As mentioned before, concentrated extracts from fresh algae were prepared in-house. Briefly, all fresh algae were freeze-dried and crushed using a grinding mill. Afterwards, 500 mg were extracted with 50% methanol solution over 30 min in an ultrasonic bath and the crude extracts were ultracentrifuged at 50,000 rpm during 20 min. Then, the supernatants were stored at −20 °C. Every supernatant was diluted 500-fold with mobile phases (5mM ammonium acetate in 90% acetonitrile) before filtration using a 0.22-µm nylon membrane syringe filter. Exposure to direct sunlight was avoided as much as possible over the sample preparation.

Methanol and acetonitrile used for extraction and analytical experiments were LC-MS grade and purchased from Honeywell (Morris Plains, NJ, USA). Ammonium acetate, acetic acid and deuterium were LC-MS grade and purchased from Sigma Aldrich (L’Isle D’Abeau Chesnes, France). Ultrapure water was obtained from a Direct-Q3 UV (Merck, Fontenay-sous-Bois, France).

#### Instrumentation

Analysis of MAAs was carried out using an Ultimate 3000 RSLC system (ThermoFisher Scientific, Bremen, Germany) coupled with an Orbitrap Fusion Lumos Tribrid mass spectrometer (ThermoFisher Scientific, Waltham, MA, USA) operated in positive mode. Fraction collection of algal extracts and infusion of MAAs were performed with a TriVersa NanoMate (Advion BioSciences, Ithaca, NY, USA) fitted on the Orbitrap Fusion Lumos instead of the standard ESI source.Data treatment for the inventory of known MAAs and characterization novel structures was carried out on Compound Discoverer 2.1^TM^ (ThermoFisher Scientific, Waltham, MA, USA). The tracking of neutral and radical losses and structural prediction of novel MAAs were carried out on Mass Frontier 7.0 ^TM^ (HighChem, Bratislava, Slovakia). 

### 2.2. Methods

#### 2.2.1. Chromatographic Conditions

The separation of MAAs was carried out on a SeQuant® ZIC-cHILIC (150 × 2.1 mm, 3 µm, 100 Å) (Merck, Fontenay-sous-Bois, France). The mobile phases were: 5mM ammonium acetate in water at pH 6.7 (A) and acetonitrile (B). The HPLC separation was carried out with the following gradient elution profile: 0–2 min, 10% B; 2–13 min, 10 to 40% B; 13–15 min, 40 to 60% B; 15–17 min, 60% B; 17–19 min, 60 to 10% B; 19–24 min, 10% B. A 20µL aliquot of diluted extract was injected.

#### 2.2.2. Untargeted Screening of MAAs

The purified MAAs palythine, porphyra-334, shinorine and mycosporine-serinol were infused at 50 ng.mL^−1^ at the flow rate 5 µL.min^−1^. Two fragmentation modes (collision-induced dissociation (CID ) and higher-energy C-trap dissociation (HCD)) were applied at different collision energies (30, 50, 70, 90 and 110). The CID parameters were: activation time at 10 ms and activation Q at 0.25. The ESI parameters were set: sheath gas at 5 (arb), auxiliary gas at 0 (arb), sweep gas at 0 (arb) and ion transfer tube temperature at 275 °C. 

**Figure 1:**
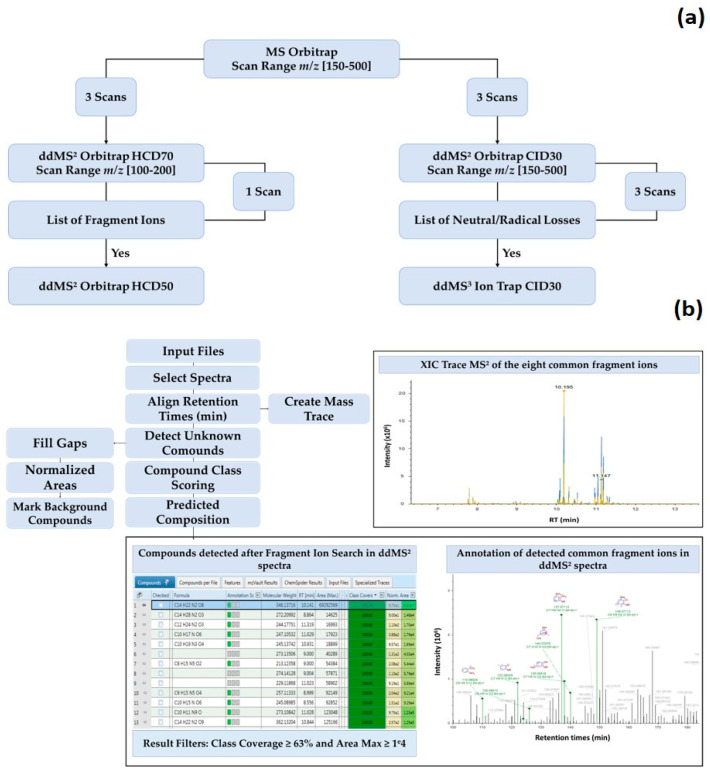
Flowchart of the developed untargeted screening approach of MAAs using an Orbitrap MS based on data-dependent MS^2^/MS^3^ Acquisition (**a**). Untargeted workflow designed on Compound Discoverer^TM^ to flag putative MAAs in algal extracts (**b**). Description of the role of the following nodes: Compound Class Scoring: Calculation of the percentage of common fragment ions detected in mass spectral data of every compound in the result table. Predicted Composition: Proposition of chemical formula for unknown compounds. Create mass Trace: Create mass chromatogram of compounds for which common fragment ions were detected in their mass spectral data. Fill Gaps: Indication of missing peaks or peaks below the detection threshold. Normalize Areas: Normalization of chromatographic peaks. Mark Background Compounds: Identification of compounds in blanks.

Figure 1a shows the scan events set in the acquisition method of the mass spectrometer. 

For LC-MS experiments, the ESI conditions were: sheath gas 50 (arb), auxiliary gas 10 (arb), sweep gas 1 (arb), ion transfer tube and vaporizer temperature 350 °C, rf lens 50% and positive ionization voltage 3500 V. Full MS Orbitrap (OT) settings were: resolution 140,000, mass range *m*/*z* 150–500, dynamic exclusion 5 s and intensity threshold 2 × 10^4^. The ddMS^2^ OT settings were: resolution 60,000 for HCD70 MS/MS scans and 30,000 for HCD50 and CID30 MS/MS scans, isolation width 2 Da. The ddMS^3^ ion-trap (IT) settings were: scan rate 33,333 Da/s, peak width ≤0.5 FWHM, isolation width 2 Da. HCD70 MS^2^ scans with a scan range set to 100–200 *m*/*z* were used to produce common fragment ions and CID30 MS^2^ was used to generate both neutral losses and small radicals. The common fragment ions were listed in the first filter permitting the triggering of further ddMS^2^ HCD50 scans for structural elucidation of candidate-MAAs. Likewise, neutral and small radical losses were included in the second filter triggering a ddMS^3^ CID30 to confirm the detection of the candidate-MAAs found in parallel with the common fragment ions. 

Compound Discoverer 2.1^TM^ software was applied to mine and identify the MAA contents in every extract in targeted and untargeted approach. The workflow illustrated in Figure 1b was designed for the untargeted analysis with a mass tolerance at 5 ppm and a minimum peak intensity at 1 × 10^4^. The retention time tolerance was fixed at 0.2 min and the signal/noise ratio at 20. To make the inventory of compounds with at least the number of elements corresponding to the skeleton core of MAAs, a minimum element threshold was defined with the molecular formula C_6_H_14_O_2_. Likewise, a maximum element threshold was set with C_90_, H_180_, O_50_, N_50_ and S_10_ to cover amino-cyclohexenones, amino-cyclohexenimines and eventually sulfated MAAs. The *Compound Class Scoring* node scores and annotates ions corresponding to the set of common fragment ions in HCD70 MS^2^ OT scans of detected compounds. A minimum number of five fragment ions were used to consider a positive MAAs flagging. The *Create Mass Trace* node plotted a XIC trace showing retention times of compounds for which the set of fragment ions were detected in HCD70 MS^2^ OT scans.

#### 2.2.3. Validation by Targeted Screening of MAAs

A Top3 MS^2^ analysis was carried out to make the inventory of MAAs in the model algal extract *Gymnogongrus devoniensis* as described in Figure S1a. The Full MS Orbitrap (OT) settings were: resolution 140,000, mass range *m*/*z* 150–500, dynamic exclusion 5 s and intensity threshold 2 × 10^4^. The MS^2^ scans settings were: HCD50 resolution 60,000, stepped collision energy +/− 20, isolation width 2 Da. For every detected mass, a FISh score was calculated to estimate the percentage of fragment ions generated in silico matching with the collected spectral data. A description of the targeted screening workflow designed on Compound Discoverer in Figure S1b.

#### 2.2.4. Identification of Novel MAAs

Fraction collection of algal extracts was performed to re-analyze by infusion the MAAs for which the structure needed to be confirmed or elucidated. The ion source of the Orbitrap Fusion Lumos was replaced by the Triversa Nanomate (on-chip nano ESI system). The latter was operated in LC chip coupling with fraction collection mode, a split setting allowing a flow of 380 nL.min^−1^ on the HD-A ESI-chip was used (remaining flow was sent to waste or to fraction collection mandrel depending on retention time). An LC coupler (Advion P/N 1003236) was used to deliver the flow on the chip. 30-s fractions were collected in a pre-washed 96 wells Advion plate from 7 to 13 min in the LC gradient. The electrospray voltage was fixed at + 1.4 kV. 

Algal extract fractions collected with the TriVersa NanoMate were re-analyzed in infusion mode by multistage fragmentation. MS^3^/MS^4^ spectra were acquired in positive mode at CID30, CID50, CID70, HCD30 and HCD50. The CID parameters were activation time at 10 ms and activation Q at 0.25. Off-line analyses of fractions were carried out with an electrospray voltage of +1.4 kV and a backing gas pressure of 0.8 psi. Ion transfer tube temperature was set at 275 °C. 

#### 2.2.5. Quantitative Analysis of MAAs

The MAAs porphyra-334, shinorine and palythine were quantified in the model algae *Gymnogongrus devoniensis* using the standard addition method. The spiked extracts were analyzed in targeted SIM (isolation width 1 min, resolution 60,000). Amounts of standardless MAAs were estimated assuming an absorption coefficient (ε) equal to the mean of the absorption coefficients of the three purified MAAs. 

## 3. Results and Discussion

### 3.1. Extraction of MAAs in Fresh Algal Samples

The polarity and the low molecular weight make MAAs readily extractable with methanol. The employed concentrations varied from 20 to 100% with controversies of the methanol concentration on the recovery [12,15,23,26]. No particular effect of the methanol concentration was observed in this study; therefore a 50% methanol solution and sonication were used to extract the MAAs from fresh algal samples. The use of ethanol proposed elsewhere [22] resulted in lower recoveries. 

### 3.2. Chromatographic Conditions

Three different stationary phases were investigated to achieve the best separation of MAAs: a reversed phase UPLC BEH C18 column (2.1 mm × 150 mm, 1.7 µm, Waters), a HILIC Kinetex HPLC column (100 × 2.1 mm, 2.6 µm, 100 Å Phenomenex) and a zwitterionic SeQuant® ZIC-cHILIC column (150 × 2.1 mm, 3 µm, 100 Å). The two latter HILIC columns differ in terms of their stationary phase one being silica diol groups and the other one functionalized silica with phosphoryl-choline groups. The optimization of the HPLC conditions was carried out by searching for the accurate mass of MAAs known in the literature in a full MS analysis of the model algae *Gymnogongrus devoniensis*. The latter was selected because of its richness of compounds. The separation performance of the three columns were compared regarding the number of MAAs efficiently retained and separated. 

**Figure 2:**
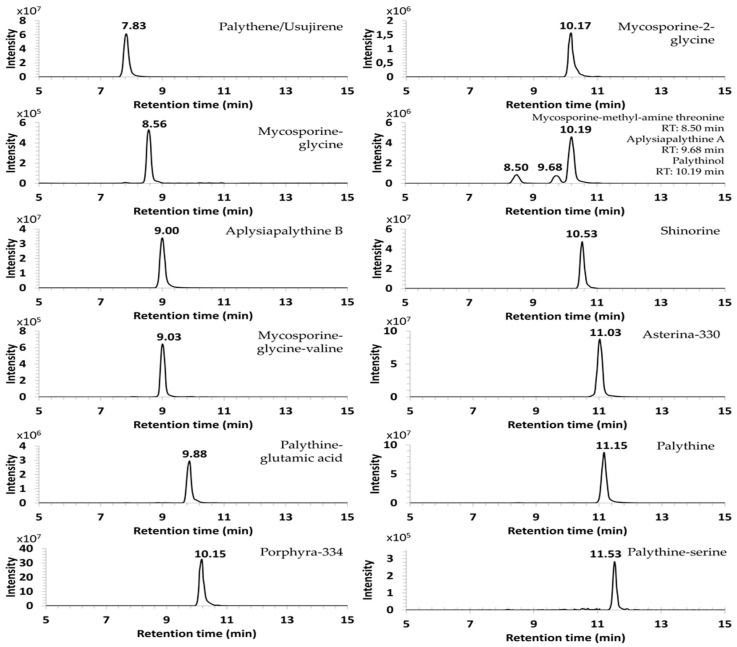
XIC of MAAs (3ppm) in Gymnogongrus devoniensis separated on the Zic-cHilic column. Palythene/Usujirene: *m*/*z* 285.1440, error −1.75 ppm; Mycosporine-glycine: *m*/*z* 246.0971, error −0.41 ppm; Aplysiapalythine B: *m*/*z* 273.1436, error −3.3 ppm; Mycosporine-glycine-valine: *m*/*z* 345.1653, error −2.06 ppm; Palythine-glutamic-acid: *m*/*z* 317.1338, error −1.58 ppm; Porphyra-334: *m*/*z* 347.1447, error −0.86 ppm; Mycosporine-2-glycine: *m*/*z* 303.1185, −0.66 ppm; Mycosporine methyl-amine threonine: *m*/*z* 303.1549, error −0.66 ppm; Aplysiapalythine A: *m*/*z* 303.1542, error −2.97 ppm; Palythinol: *m*/*z* 303.1548, error −0.66 ppm; Shinorine: *m*/*z* 333.1286, error −1.8 ppm; Asterina-330: *m*/*z* 289.1389, −1.73 ppm; Palythine: *m*/*z* 245.1123, error −2.85 ppm; Palythine-serine: *m*/*z* 275.1234, error −1.45 ppm.

The first results showed the MAAs were not retained on the UPLC BEH C18 column using a gradient elution with 0.1% of formic acid in acetonitrile/water. The chromatographic method proposed elsewhere [12] to separate strongly acidic, neutral MAAs and the isomeric couple cis-usujirene and trans-palythene using acetonitrile-based eluents and polymeric double endcapped C18 columns turned out to be insufficient to separate highly polar compounds. The use of the HILIC Kinetex HPLC column improved the retention of high polar MAAs on the stationary phase but did not allow a satisfactory resolution and symmetric peaks. An alternative method based on the zwitterionic properties of MAAs using a Zic-HILIC column improved their separation [15]. As is shown in Figure 2, 14 MAAs were efficiently retained on the SeQuant® ZIC-cHILIC column and identified in the full MS scan with a mass error lower or equal to 3 ppm. The same separation method was performed on four other algal extracts for which MAA-profiles were detailed in Figure S2a–d.

The separation efficiency of MAAs and their ionization conditions were optimized using ammonium acetate solution at 5 mM and pH 6.5 [14,15]. In these conditions, 14 candidate-MAAs were separated between 7 and 12 min. 

As it was discussed elsewhere, coelution was observed in almost all the separation procedures of MAAs owing to the presence of several isomers. Moreover, the disparity of their polarity made it more difficult to choose an efficient column retaining all the MAAs without coelution [10,12]. With the increasing number of MAAs accounted for, coelutions cannot be avoided and the HRAM MS detection, which allows the resolution of all the MAAs except the mass isomers, is mandatory. The separation of isomers has to be addressed by multistage MS^n^ analysis. 

### 3.3. Identification of Cancidate-MAAs in Untargeted Analysis 

#### 3.3.1. Fragmentation Pathways of MAAs

The goal was to investigate the fragmentation patterns of MAAs in order to define a set of characteristic fragment ions, neutral and radical losses to serve the untargeted detection of MAAs and to find the optimal conditions to generate these fragments. For this purpose, several levels of collision energies were tested on three purified imino-MAAs included porphyra-334, shinorine, palythine and one oxo-MAA, mycosporine-serinol. Porphyra-334, shinorine and palythine belong to the palythine and glycine families considered to be the most common in marine algae [7,9]. The typical MAA of lichens—mycosporine-serinol—was used as a fragmentation model of oxo-MAAs. 

**Figure 3:**
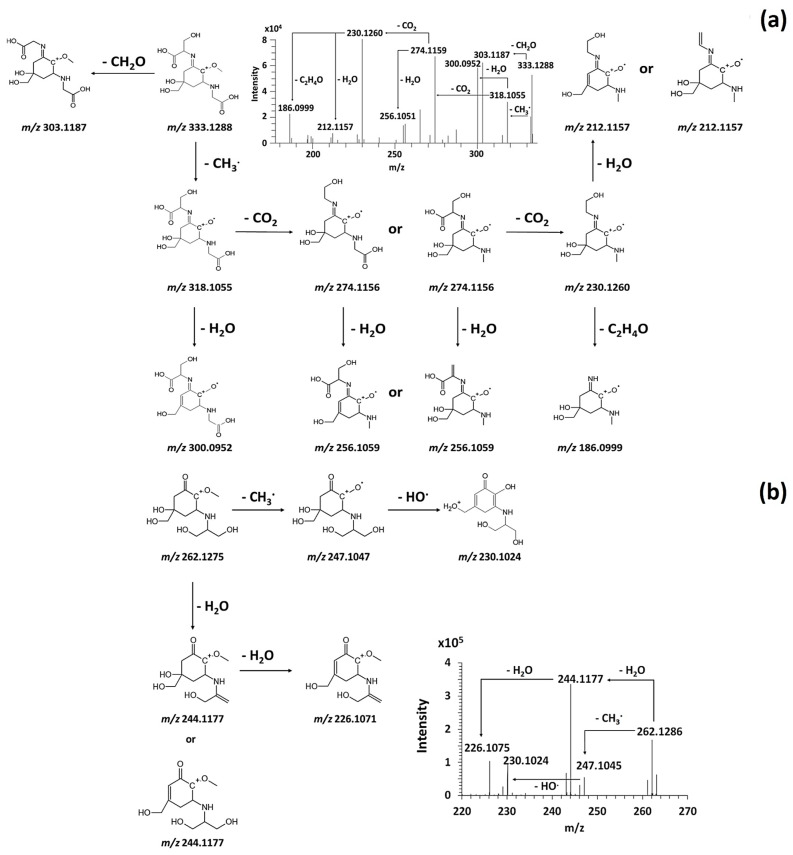
Fragmentation pathways of shinorine (**a**) and mycosporine-serinol (**b**) from the fragmentation data collection of their CID30MS^2^ spectrum.

The study of the CID30 MS^2^ spectra of the purified MAAs permitted to highlight in Figure 3 their radical fragmentation triggered by the weakening of the ether bond C-O and the loss of the methyl radical (15,023 Da) [26,27]. 

Successive decarboxylation or dehydration were mainly observed on the [(M+H) - CH_3_] ^+**.**^ MAA moiety according to the structure of substituents on the C_1_ and C_3_ [24,26,28]. The fragmentation of palythine resulted mainly in the loss of water (18.010 Da) and carboxyl (43.989 Da) on the glycine group producing the last most intense fragment ion *m*/*z* 186.0999 (Figure S3a). This fragment ion was also produced after the dealkylation of *m*/*z* 230.1260 (C_2_H_4_O, 44.026 Da) and *m*/*z* 244.1417 (C_3_H_6_O, 58,041 Da) in CID30 MS^2^ spectra of shinorine (Figure 3a) and porphyra-334 (Figure S3b). Notably, the CID30 MS^2^ spectrum of shinorine and porphyra-334 exhibited a very low intense methyl radical loss and showed highly intense [(M + H)−CH_2_O] ^+^ and [(M + H)−C_2_H_4_O] ^+^ moieties instead, corresponding to the same fragment ion *m*/*z* 303.1187 (Figure 3a and Figure S3b). Indeed, the loss of formaldehyde was observed in prior positive ESI-MS^2^ analysis of shinorine. On the contrary, the monitoring of the fragmentation in HRMS^n^ analysis has demonstrated the fragment ion *m*/*z* 303.1187 produced from the porhyra-334 came from a dealkylation (44.026 Da) and not from a decarboxylation (43.989 Da) as it was suggested in prior low-resolution mass spectrometry studies (Figure S3b). As it was demonstrated formerly, the presence of two di-acidic functions in MAA structure could modify the driving force of the fragmentation to give priority to decarboxylation [27,29,30]. Similarly, the presence of the ketone group and/or the atypical serinol group of the mycosporine-serinol could also give the priority to dehydration in its fragmentation pathway as shows the CID30 MS^2^ spectrum in Figure 3b. Nonetheless, the fragment ion *m*/*z* 247.1049 obtained after the radical methyl loss underwent a rearrangement of its carbon centered radical structure by resonance releasing a hydroxide radical loss and producing the fragment ion *m*/*z* 230.1024. This observation assumed a likely competition between fragmentation pathways starting either with the radical methyl or water loss. Thus, the CID30 MS^2^ spectra of purified MAAs allowed the inventory of the most characteristic neutral and radical losses useful in the monitoring of their different fragmentation pathways and their mining in untargeted analysis.

**Figure 4:**
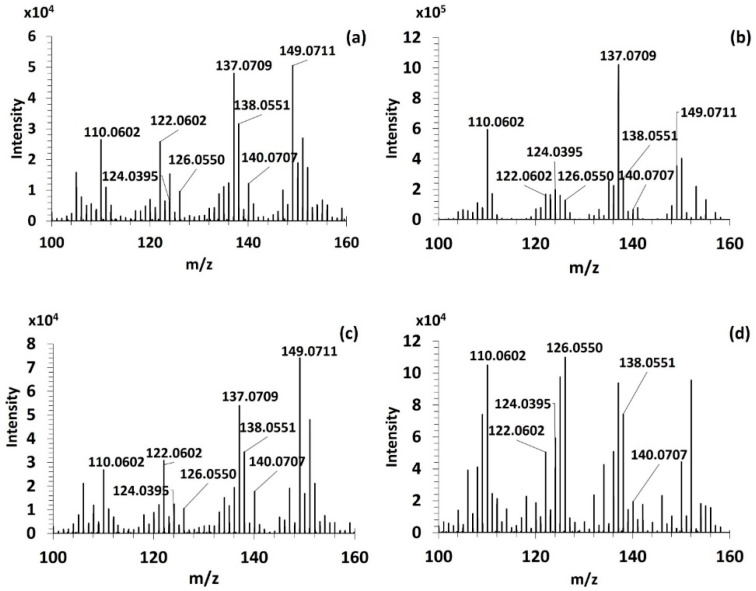
HCD70 MS^2^ spectrum of shinorine (**a**), palythine (**b**), porphyra-334 (**c**) and mycosporine-serinol (**d**).

Although the fragmentation at CID30 permitted to identify the fragment ion *m*/*z* 186.0999 specific of imino-MAAs [28,30], no common fragment ion was detected between imino- and oxo-MAAs. Indeed, low collision energies permitted only to describe the first steps of their radical fragmentation pathway targeting exclusively their substituents. An optimization of the fragmentation conditions at HCD70 allowed to produce, as it is shown in Figure 4 six common fragment ions among the four purified MAAs: *m*/*z* 110.0602, *m*/*z* 122.0602, *m*/*z* 124.0395, *m*/*z* 126.0550, 138.0551 and *m*/*z* 140.0707. Besides, mass spectral data of the three imino-MAAs showed two characteristic high intense fragment ions: *m*/*z* 137.0709 and *m*/*z* 149.0711 (Figure 4a–c).

These fragment ions were distributed in the *m*/*z* 100–140 range in the spectra of the purified MAAs (Figure 4a–d). The fragment ions found in this mass range lost not only all the substituents but also the ketone or imino-group on the C_1_ which distinguish oxo- and imino-MAAs. This hypothesis permitted to justify the significative differences observed in the fragment ion distribution above *m*/*z* 140 in the mass spectral data of imino- and oxo-MAAs. An exception was observed for the fragment ions *m*/*z* 137.0709 and *m*/*z* 149.0711 in HCD70 MS^2^ only found in imino-MAA spectra (Figure 4a–c). Of note, shinorine and porphyra-334 displayed the same fragmentation tendency due to their similar structure differing only by an additional methyl group on porphyra-334 (Figure 4a,c). Moreover, the fragment ion distribution above *m*/*z* 160 in a HCD70 MS^2^ spectrum of palythine (Figure 4b) differed from those observed in spectra of porphyra-334 and shinorine because of the absence of substituent on the C_1_. 

**Figure 5:**
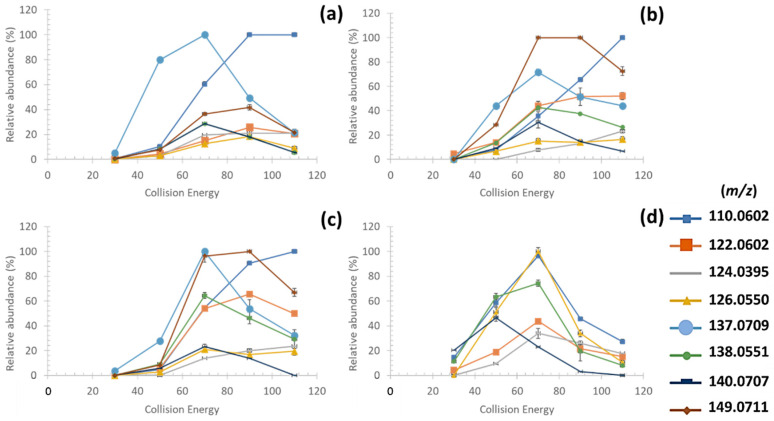
Relative abundance of characteristic fragment ions of MAAs depending on collision energies in MS^2^ of palythine (**a**), porphyra-334 (**b**), shinorine (**c**) and mycosporine-serinol (**d**).

The comparison between different fragmentation conditions revealed that the greatest relative abundance of the six common fragment ions among oxo- and imino-MAAs was achieved at HCD70 as it is shown in Figure 5. 

The fragment ion *m*/*z* 110.0602 showed high intensity in all HCD70 MS^2^ spectrum of MAAs (Figure 5a–d) which supposed a common fragmentation route for any MAA regardless of the MAA class. Finally, the fragment ions at *m*/*z* 137.0709 and *m*/*z* 149.0711 displayed high intensities in HCD70 MS^2^ spectra of imino-MAAs (Figure 5a–c), which confirmed their relevance for the distinction between imino- and oxo-candidate-MAAs detected in algal extracts as part of the untargeted analysis. 

On the basis of the monitoring of the fragmentation of imino- and oxo-MAAs, a set of eight fragment ions including: *m*/*z* 110.0602, *m*/*z* 122.0602, *m*/*z* 124.0395 and *m*/*z* 126.0550, *m*/*z* 137.0709, *m*/*z* 138.0551, *m*/*z* 140.0707 and *m*/*z* 149.0711 were selected to develop an untargeted screening approach for MAAs in algal extracts.

#### 3.3.2. Mining and Annotation of MAAs in Algal Model Extract

An untargeted ddMS^2^/MS^3^ analysis was carried out using the set of eight fragment ions, neutral and radical losses defined above to screen for candidate-MAAs in the model algae *Gymnogongrus devoniensis.*

Data-processing using the untargeted workflow designed using Compound Discoverer^TM^ software to mine and flag candidate-MAAs retrieved 1498 candidate compounds. The detection of MAAs was considered positive when the intensity of precursor ions exceeded the 1 × 10^4^ threshold and when a minimal number of five fragment ions out of eight could be observed in their HCD70 MS^2^ OT scans. These criteria of result selection reduced the number of candidate-MAAs to 41 as reported in Table 3. 

All the 41 exact masses were listed in the final result table according to the number of fragment ions detected in their HCD70 MS^2^ OT scans. Of note, 14 of them were identified in the literature [26].

The annotation of candidate-MAA was also carried out with the detection of characteristic neutral and radical losses. Indeed, a CID30 ddMS^3^ IT scan was triggered after the detection of neutral and radical losses in prior CID30 ddMS^2^ OT scan of every putative MAAs. Mechanisms of decarboxylation and dehydration were observed on the [(M+H)-CH_3_] ^+.^ moiety in the putative MAA spectra as it was detailed in Figure S4. These observations proved the tracking of neutral and radical losses was all the more relevant since the fragmentation pathways of MAAs permitted the production of the eight characteristic fragment ions observed in ddMS^2^ HCD70 OT spectra. In this way, any detected mass showing a CID30 ddMS^3^ IT scan in addition to a HCD70 ddMS^2^ OT scan with at least five annotated fragment ions was a putative MAA. 

Surprisingly, four exact masses in Table 3 coeluted with the identified MAAs palythene/usujirene (225,1237 Da), asterina-330 (229,1187 Da; 273,1084 Da) and palythine (185,0924 Da). The detected mass 185.0924 Da corresponded to the fragment ion *m*/*z* 186.099 which gave origin to all the fragment ions characteristic of imino-MAAs. Likewise, the detected masses 229.1187 Da and 273.1084 Da have been already reported in the literature as typical fragment ions in the fragmentation pathways of asterina-330 [28]. These observations indicated in-source fragmentation of palythene/usujirene, asterina-330 and palythine. Moreover, they confirmed the capability of the method developed to retrieve coeluted MAAs. 

In summary, the untargeted screening approach permitted to flag successfully 23 novel candidate-MAAs in *Gymnogongrus devoniensis* in addition to the 14 known ones. Besides, these findings corroborated the hypothesis that this red alga contained a high diversity in MAAs characteristic of algae belonging to the Bangiales order [21]. 

### 3.4. Method Validation by Targeted Analysis

To validate the developed untargeted screening method, a targeted analysis based on a Top3 MS^2^ acquisition was carried out on the model algal extract *Gymnogongrus devoniensis*. The workflow designed for the targeted screening of MAAs illustrated in Figure S1. 

All the detected compounds were searched for in the in-house database of over 41 reported MAAs. At the outcome of the targeted screening analysis, 14 compounds were detected and identified on Table 4. 

All the exact masses were detected with 3 ppm mass accuracy. The FISh score showed at least 50% of experimental fragment ions detected in spectra of 9 MAAs matched with those found in silico. The stepped collision-energy mode was applied to collect and compile mass spectral data both in HCD30 and HCD70 permitting thus the detection of the set of eight fragment ions, neutral and radical losses chosen in untargeted analysis. Fragmentation at HCD30 allowed the production of intense fragment ions showing the first fragmentation steps of MAAs involving demethylation, decarboxylation and dehydration (Table 4, See also Figure S5). Only the mycosporine-glycine did not display the fragment ions *m*/*z* 137.0709 and *m*/*z* 149.0711 confirming its affiliation to the oxo-MAAs. Similarly, the fragmentation permitted to make the distinction between the palythine-glutamic acid newly characterized by *Orfanoudaki* et al. [16] and its oxo-isomer mycosporine-glutamine in the algal sample. Hence, the choice of the fragment ion set to mine candidate-MAAs allowed a satisfactory coverage of their structural diversity.

In conclusion, the targeted analysis of *Gymnogongrus devoniensis* permitted to validate the developed untargeted ddMS^2^/MS^3^ analysis and confirm its efficiency to cover the MAA present in a sample.

### 3.5. Discovery of Novel MAAs

Fractions resulted from the HILIC fractionation of the extract of *Gymnogongrus devoniensis* were collected on-line in order to elucidate, or confirm by multistage fragmentation, the structures of seven candidate-MAAs detected in untargeted analysis.

As it is shown in Table 5, the study of the ion precursors and their most intense fragment ions in multistage fragmentation permitted to complete the fragmentation data obtained in untargeted analysis.

MAAs for which CID30 MS^2^ mass spectral data were not sufficient for the structural elucidation were studied using MS^3^ and MS^4^ fragmentation, which completed the description of their fragmentation routes. 

**Figure 6:**
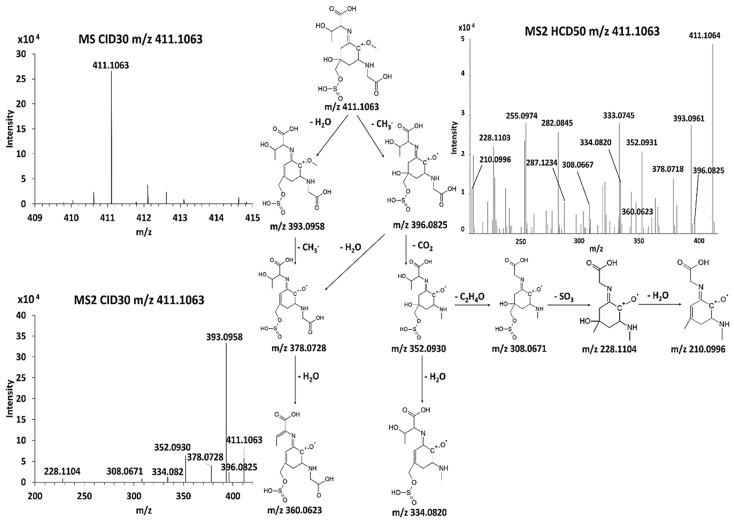
Structural elucidation of the novel MAAs *m*/*z* 411.1063 based on MS^3^/MS^4^ data collection.

Three main fragmentation routes starting either with a methyl radical loss, decarboxylation or dehydration were observed in most mass spectral data of elucidated MAAs. As it was illustrated in Figure 6, the multistage fragmentation of the novel MAA *m*/*z* 411.1063 permitted to propose a putative structure by monitoring the fragment ions produced in two main fragmentation pathways.

The most intense fragment ion observed in the CID30 MS^2^ spectrum of the candidate-MAA *m*/*z* 411.1063 was *m*/*z* 393.0958. This result supported the hypothesis that the radical elimination pathway, specific to the MAAs, could compete with secondary fragmentation routes. The priority choice of a given fragmentation pathway depends on the presence and the number of functional groups on their amino acids substituents such as hydroxyl or carboxylic groups [29]. The observation of the neutral loss SO_3_ (79.95 Da) suggested for the first time the occurrence of sulfated MAAs. This neutral loss was also observed for the candidate-MAA *m*/*z* 367.1164. The multistage fragmentation pathways of six other MAAs were detailed in Figure S6a–f.

The multistage fragmentation allowed the attribution to MAAs of hypothetical structures which are given in Table 6. 

In particular, the fragmentation data allowed the identification of the functional substituents. Indeed, the R-group could be predicted on the structural modifications of the most common amino acids found in algal MAAs including decarboxylation, dehydration, carbonyl reduction and substitutions. This approach permitted to propose the classification of the eight candidate-MAAs into the palythine family, and the threonine, serine and alanine subfamilies [9]. The limitation of this assignment is the impossibility of the identification of the isomers present. Hence, more than one likely structure could be assigned for candidate-MAAs mentioned in Table S1. The detection of unknown MAAs is based on the fact that they have very similar chemical structures and produce many identical fragment ions. Therefore, the precise structure assignment may not be straightforward. The structural differences can be very small and the set of MS^n^ fragment ions, as complete as it could be, may not be enough to propose unambiguous structures for all the MAAs detected, especially that many isomers occur. For instance, several structures can be proposed for their substituents on C1 and C3 carbons and imino and oxo-MAA isomers are common. To assure the highest quality of data interpretation, it is necessary to carry out a multistage fragmentation at different collision and energy modes to produce a larger set of fragment ions and distinct every isoform regarding the presence of different fragment ions. For every isomer, a FISh score was calculated to determine the highest percentage of fragment ions matching with those found in silico at a specific retention time. This permitted to suggest the most probable isomer. Concerning imino and oxo-MAA isomers, the latter can be easily distinguished by the detection of two characteristic fragment ions appearing specifically in imino-MAA MS^2^ HCD70 spectrum. 

Please note that because of the possible coelution of different candidate-MAAs, isolation and sufficient purification of them could not be achieved to confirm the structures by NMR.

Therefore, LC using chiral stationary phases have become an essential tool in the determination of enantiomeric composition in complex samples [31]. The identification of one to several chiral centers on amino acid substituents or their derivatives in MAA structure could help to locate specific functional groups in their lateral chains. Nonetheless, chiral chromatography favored normal-phase using solvents incompatible with ESI interface in LC-MS^n^ applications. Although the increasing interest in developing chiral columns more versatile, the solvent and pH extraction and mobile phases turned out to be limiting factors in the identification of stereoisomers because of the possible enantioselective ion suppression or enhancement in ESI-MS analysis [32]. Moreover, most of the current analytical studies conducted still consist of targeted screening of enantiomeric compounds [31]. Hence the requirement to optimize analytical procedures to improve the characterization of stereoisomers with a broad range of physico-chemical properties in untargeted LC-MS^n^ analysis [33].

### 3.6. Quantification

**Figure 7:**
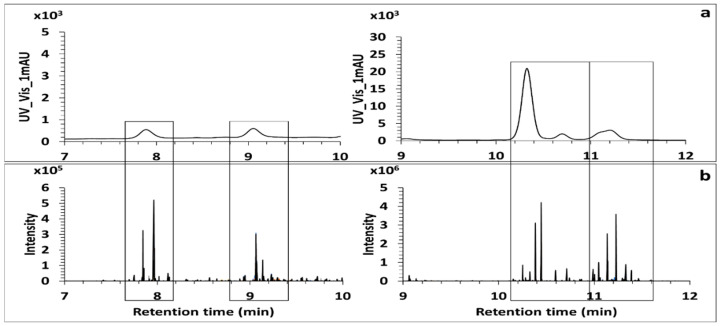
Screening of MAAs in the model alga *Gymnogongrus devoniensis*. UV Trace of MAAs recorded at (300–360) nm (**a**). XIC Trace MS^2^ of the set of eight fragment ions by ddMS^2^/MS^3^ untargeted analysis (**b**). All the peaks observed in the XIC trace MS^2^ indicate all the retention times for which MAAs were detected in the extract.

Quantification of MAAs in algal extracts has usually been performed according to their molar extinction coefficient in HPLC-DAD [13,18,20] or by HPLC-MS [14,29] analysis making use of their MS signal and matching it with standards. As it is shown in Figure 7, analysis of the model algal extract *Gymnogongrus devoniensis* by HPLC with DAD detection revealed five major peaks in the UV trace. 

These five peaks covered all the peaks observed in the XIC trace of the eight fragment ions for which retention times corresponded to retention times of MAAs detected in untargeted screening analysis. These results indicate that the HPLC-DAD resolution is insufficient to quantify the individual species and the results reported by these methods may suffer from the contribution of other MAAs than those targeted by the authors [20,34]. HPLC- MS/MS would be specific enough for quantification but as the sensitivity is a function of ionization, authentic standards of quantified MAAs are required, and they are not available.

It has to be emphasized that except for porphyra-334, shinorine and palythine for which calibration standards were available, and which were quantified by the method of standard additions, the results in Table 7 are purely estimative, based solely on the hypothesis that the electrospray MS response factor would be similar (within 10%) for all the MAAs, as it was demonstrated for the three MAAs for which the standards were available. 

Interestingly, porphyra-334 and palythine were the most abundant in the model algae *Gymnogongrus devoniensis* as previously reported for algal species belonging to the Bangiales order [35]. Please note that an isolation process of shinorine was developed from this red algal specie [20].

## 4. Applications

The method developed was applied on three red algae (*Gelidium sesquipedale*, *Halopithys incurva* and *Porphyra rosengurtii*) and one brown alga (*Cystoseira tamariscifolia*) allowing the detection of tens MAAs reported in the literature, three MAAs detected and characterized here in the model alga *Gymnogongrus devoniensis* and four more novel candidate-MAAs reported on Table 8.

At least seven putative MAAs were detected in every red algal species. This was in contrast with the brown alga *Cystoseira tamariscifolia* in which only three MAAs were found, which confirms the specificity of the MAA synthesis in Rhodophyta taxa [34]. Notably, the 284.1369 Da mass was detected at different retention times (7.8–9.3min) in the alga *Halopithys incurva* suggesting the presence of both palythene and usujirene. However, their discrimination using tandem mass spectrometry or DAD detection remains limited due to their very close structures and molar extinction coefficients.

## 5. Conclusions

A set of eight fragment ions after data-dependent MS^2^/MS^3^ acquisition are a reliable bases for untargeted screening of mycosporines and MAAs in algal extracts. The developed HRAM-ESI-MS^n^ method permitted to discover unreported compounds and widen the MAA profiling of five algae regardless of their UV-absorbing properties without referring to existing databases or standards. Additional multistage fragmentation after fraction collection of algal extracts allowed the characterization of seven unreported MAAs without requiring their purification. The study reaches the limits in terms of the assignment of isomeric MAAs structures by the state-of-the-art MS technology. One of the upcoming challenges beyond this state-of-the-art is the identification of MAA stereoisomers. Indeed, MAAs have some (1-3) chiral carbons and may occur as many stereo-isomers with specific properties. The method responds to the increasing interest in the attribution to an algal MAA-profile for commercial health and cosmetic formula and bio-inspired materials. 

## Figures and Tables

**Table 1:** Classification of mycosporines, MAAs and their precursor reported in the literature. The structure and the theoretical monoisotopic mass (Da) were detailed for every compound classified in five families depending on their amino acid substituents on the C3. The structural relationships were briefly described for every example [9].

MAA Family	Precursor	Serine Family	Glutamine Family	Palythine Family	Glutamic Acid Family
Name	4-deoxygadusol (4-DG)	Mycosporine-serinol (M-SerOH)	Mycosporine-glutamine (M-Gln)	Palythine (PNE. M-NH2:Gly )	Mycosporine-glutamic acid (M-Glu)
Structure	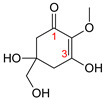	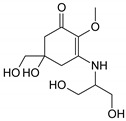	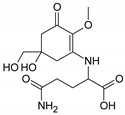	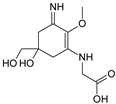	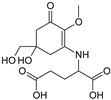
Monoisotopic mass (Da)	188.0685	261.1212	316.1271	244.1059	317.1111
Structural relationships	Reduction of Gadusol	Reduction of M-Ser	4-DG + Gln→ M-Gln + H_2_O	4-DG + NH_2_ + Gly → M-NH_2_:Gly + H_2_O	4-DG + Glu → M-Glu + H_2_O
**MAA Family**	**Glycine Family**				
**Subfamily**	**Glycine** **Subfamily**	**Serine** **Subfamily**	**Valine** **Subfamily**	**Threonine Subfamily**	**Alanine** **Subfamily**
Name	Asterina-330 (Ast. M-Gly:Gly(OH))	Shinorine (SH) (M-Gly:Ser)	Mycosporine-glycine-valine (M-Gly:Val)	Porphyra-334 (P-334. M-Gly:Thr)	Palythinol (Pinol) (M-Gly:Ala(OH))
Structure	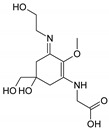	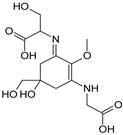	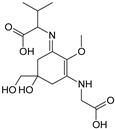	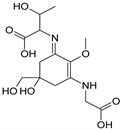	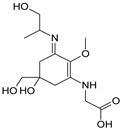
Monoisotopic mass (Da)	288.1321	332.1219	344.1583	346.1376	302.1478
Structural relationships	Reduction of M-2Gly	M-Gly + Ser→ M-Gly:Ser + H_2_O	M-Gly + Val→ M-Gly:Val + H_2_O	M-Gly + Thr→ M-Gly:Thr + H_2_O	M-Gly + Ala→ M-Gly:Ala + H_2_O (2) Reduction of M-Gly:Ala

**Table 2:** Inventory of MAAs identified in targeted HPLC-ESI-MS methods operated in positive mode.

Marine Organisms	Number of MAAs	MAAs	HPLC Conditions	MS Analysis	Literature
*Brostrychia scorpioides. Porphyra dioica. Gracilaria vermiculophylla. Vertebrata lanosa* (Red Algae)	6	Shinorine. palythine. asterina-330. porphyra-334. usijirene. palythene.	Reversed-phase chromatography ACE C18 column (150 × 4.6 mm; 3 μm)	Q-TOF Resolution 20.000 FWHM Full MS scan range: *m*/*z* 50–1000	Lalegerie et al. (2019) [22]
*Rhodymenia pseudopalmata* (Red Alga)	8	Deoxygadusol. porphyra-334. shinorine. palythine. asterina-330. palythinol. usijirene. palythene.	Reversed-phase chromatography Luna Omega C18 (250 × 4.6 mm; 5 µm)	Q-TOF Resolution 20.000 FWHM Full MS scan range: *m*/*z* 100–1000	Pliego-Cortès et al. (2019) [24]
*Oscilatoria* sp. (Cyanobacteria)	13	Palythine. shinorine. porphyra-334. palythine-serine. palythine threonine. mycosporine-glycine. mycosporine-taurine. mycosporine-ornithine. hexose-bond palythine-serine and hexose-bond palythine-threonine.	Reversed-phase chromatography Synergi Hydro-RP 80A column (150 × 2.0 mm; 4 μm)	Q-TOF Resolution 20.000 FWHM Full MS scan range: *m*/*z* 50–500 MS/MS: Top3 MS^2^	Geraldes et al. (2019) [23]
*Catenella repens.* (Red Alga)	1	Catenelline	HILIC chromatography Sequant ZIC-HILIC (250 mm × 4.6 mm; 5 μm)	Q-TOF Ion-trap Full MS scan range: *m*/*z* 100–1500	Hartmann et al. (2015) [15]
Microalgae	5	Palythene. palythine. mycosporine-glycine. palythenic-acid. porphyra-334. shinorine.	Normal-phase chromatography Luna NH2 column (250 × 4.6 mm; 5 μm)	Ion-trap MS^3^ scans	Llewellyn and Airs (2010) [13]
*Palmaria palmata* (Red Alga)	6	Palythine. shinorine. asterina-330. palythinol. porphyra-334. Usujirene	Reversed-phase chromatography Inertsil ODS-3 column (250 mm × 4.6µm; 5 µm)	Ion-trap MS scan: *m*/*z* 50-600	Yuan et al. (2009) [21]
*Pocillopora capitata* (Coral)	12	Palythine-serine-sulfate. mycosporine sulfate. shinorine. mycosporine-2-glycine. palythine-serine. palythine. porphyra-334. mycosporine-methylamine-serine. mycosporine-glycine. palythine-threonine. palythinol. mycosporine-methylamine-threonine.	Reversed-phase chromatography C18 column (4.6 × 150 mm; 3 µm)	Ion-trap. TOF Resolution 10.000 MS^2^/MS^3^ scans	Carignan et al. (2009) [18]

**Table 3:** Candidate-MAAs detected in the model algae *Gymnogongrus devoniensis* using the Fragment Ion Search (FISh).

MAAs	In Source Fragmentation	Formula	Monoisotopic Mass (Da)	[M+H]+ (*m*/*z*)	Area Max (10^5^)	RT [min]	Number of Fragment Ions (/8)
**Porphyra-334**	-	**C_14_ H_22_ O_8_ N_2_**	**346.1372**	347.1444	693	**10.14**	**8**
Unknown	-	C_14_ H_28_ O_3_ N_2_	272.2099	273.2170	0.15	8.86	8
Unknown	-	C_10_ H_17_ O_6_ N	247.1053	248.1126	0.18	11.03	8
Unknown	-	C_10_ H_19_ O_4_ N_3_	245.1374	246.1447	0.19	10.93	8
Asterina-330	[(M+H)-(CH_3_; CO_2_)]	C_10_ H_17_ O_4_ N_2_	229.1187	230.1259	0.59	11.02	8
Asterina-330	[(M+H)-CH_3_]	C_11_ H_17_ O_6_ N_2_	273.1084	274.1157	1.23	11.03	8
Unknown	-	C_14_ H_22_ O_9_ N_2_	362.1320	363.1389	1.25	10.84	8
Palythine	[(M+H)-(CH_3_; CO_2_)]	C_8_ H_13_ O_3_ N_2_	185.0924	186.0999	1.02	11.15	8
Unknown	-	C_12_ H_19_ O_3_ N_3_ S	285.1142	286.1215	0.10	8.11	8
**Aplysiapalythine-B**	-	**C_12_ H_20_ O_5_ N_2_**	**272.1367**	273.1436	76.5	**9.00**	**8**
**Shinorine**	-	**C_13_ H_20_ O_8_ N_2_**	**332.1214**	333.1292	85.2	**10.52**	**8**
**Palythinol**	-	**C_13_ H_22_ O_6_ N_2_**	**302.1475**	303.1551	11.3	**10.20**	**8**
**Mycosporine-2-glycine**	-	**C_12_ H_18_ O_7_ N_2_**	**302.1112**	303.1185	3.55	**10.15**	**8**
**Palythine**	-	**C_10_ H_16_ O_5_ N_2_**	**244.1051**	245.1124	122	**11.14**	**8**
Unknown	-	C_15_ H_24_ O_10_ N_2_ S	424.1146	425.1218	4.73	10.16	8
**Mycosporine methyl-amine threonine**	-	**C_13_ H_22_ O_6_ N_2_**	**302.1475**	303.1551	2.43	8.48	**8**
**Asterina-330**	-	**C_12_ H_20_ O_6_ N_2_**	**288.1316**	289.1394	187	**11.03**	**8**
**Palythene/Usujirene**	-	**C_13_ H_20_ O_5_ N_2_**	**284.1368**	285.1445	137	**7.81**	**8**
Palythene/Usujirene	[(M+H)-(CH_3_; CO_2_)]	C_11_ H_17_ O_3_ N_2_	225.1237	226.1311	0.39	7.81	7
Unknown	-	C_13_ H_22_ O_7_ N_2_	318.1425	319.1493	1.63	10.17	7
Unknown	-	C_13_ H_20_ O_6_ N_2_	300.1320	301.1392	1.99	10.15	7
Unknown	-	C_11_ H_18_ O_7_ N_2_ S	322.0827	323.0899	0.58	11.15	7
Unknown	-	C_11_ H_18_ O_5_ N_2_	258.1217	259.1289	1.07	10.05	7
**Mycosporine-glycine-valine**	-	**C_15_ H_24_ O_7_ N_2_**	**344.1579**	345.1652	1.20	**9.01**	**7**
Unknown	-	C_14_ H_22_ O_7_ N_2_	330.1420	331.1491	1.07	9.43	7
**Palythine glutamic-acid**	-	**C_13_ H_20_ O_7_ N_2_**	**316.1266**	317.1338	6.26	**9.86**	**7**
Unknown	-	C_20_ H_30_ O_10_ N_2_	458.1903	459.1975	2.77	10.04	6
Unknown	-	C_13_ H_22_ O_8_ N_2_ S	366.1092	367.1164	0.49	11.02	6
**Palythine-serine**	-	**C_11_ H_18_ O_6_ N_2_**	**274.1158**	275.1231	0.47	**11.54**	**6**
Unknown	-	C_16_ H_26_ O_8_ N_4_	402.1751	403.1816	0.44	10.69	6
Unknown	-	C_15_ H_24_ O_8_ N_2_	360.1522	361.1602	0.28	9.52	6
Unknown	-	C_13_ H_23_ O_6_ N_5_	345.1647	346.1722	0.22	11.91	5
Unknown	-	C_15_ H_24_ O_8_ N_2_	360.1522	361.1598	0.21	9.96	5
Unknown	-	C_13_ H_22_ O_5_ N_2_	286.1525	287.1240	0.17	8.05	5
Unknown	-	C_15_ H_23_ O_8_ N_3_	373.1484	374.1557	0.53	10.50	5
Unknown	-	C_18_ H_22_ O_8_ N_2_	394.1369	395.1441	0.46	8.28	5
Unknown	-	C_14_ H_22_ O_10_ N_2_ S	410.0990	411.1060	0.50	10.53	5
Unknown	-	C_13_ H_20_ O_6_ N_2_	300.1320	301.1392	2.66	9.28	5
**Aplysiapalythine-A**	-	**C_13_ H_22_ O_6_ N_2_**	**302.1475**	303.1551	2.07	**9.72**	**5**
Unknown	-	C_14_ H_22_ O_7_ N_2_ S	362.1143	363.1215	0.28	7.81	5
**Mycosporine-glycine**	-	**C_10_ H_15_ O_6_ N**	**245.0899**	246.0972	0.93	**8.56**	**5**

The known MAAs were emphasized in bold. MAAs subject to ion source fragmentation were indicated by the moiety [(M+H)-neutral and/or radical loss] ^+^ generated.

**Table 4:** Targeted analysis of the model algal sample *Gymnogongrus devoniensis*. FISh coverage indicates the percentage of experimental fragment ions matching with those obtained in silico fragmentation. The number of fragment ions included in the set used in untargeted analysis of MAAs is also referred. * Please note that mycosporine-glycine was the only compound for which the fragment ions *m*/*z* 137.0709 and *m*/*z* 149.0711 were not detected in ddMS^2^ spectrum.

MAAs	[M+H]^+^	Retention Time (min)	FISh Coverage (%)	Number of Characteristic Fragment Ions of MAAs (/8)	Neutral and Radical Losses
Palythine	245.1123	11.14	42	8	CH_3_; CO_2_; H_2_O
Mycosporine-glycine *	246.0971	8.56	69	5	CH_3_; CO_2_; H_2_O
Aplysiapalythine B	273.1436	9.01	52	8	CH_3_; CO_2;_ H_2_O
Palythine-serine	275.1234	11.54	67	5	CH_3_; CO_2_; H_2_O
Palythene /Usujirene	285.1440	7.81	48	8	CH_3_; CO_2_; H_2_O
Asterina-330	289.1389	11.03	59	8	CH_3_; CO_2_; H_2_O CH_3_O; C_2_H_4_O
Mycosporine-2-glycine	303.1185	10.15	50	8	CH_3_; CO_2_; H_2_O
Aplysiapalythine A	303.1542	9.72	38	4	CH_3_; CO_2_; H_2_O
Palythinol	303.1548	10.20	57	8	CH_3_; CO_2_; H_2_O
Mycosporine-methyl amine threonine	303.1549	8.48	64	8	CH_3_; CO_2_; H_2_O
Palythine-glutamic acid	317.1338	9.86	45	8	CH_3_; CO_2_; H_2_O
Shinorine	333.1286	10.52	48	8	CH_3_; CO_2_; H_2_O; CH_2_O; C_2_H_4_O
Mycosporine-glycine-valine	345.1653	9.03	63	7	CH_3_; CO_2_; H_2_O
Porphyra-334	347.1444	10.14	67	8	CH_3_; CO_2_; H_2_O; C_2_H_4_O; C_3_H_6_O

**Table 5:** Structural elucidation of novel MAAs. Fragment ions detected in MS^2^, MS^3^ and MS^4^ fragmentation were reported for the seven masses annotated from A–G.

Compound	[M+H]^+^ (*m*/*z*)	Formula [M+H]^+^	Fragment Ions (*m*/*z*)		
			MS^2^	MS^3^	MS^4^
A	301.1393	C_13_ H_21_ O_6_ N_2_	286.1158 C_12_ H_18_ O_6_ N_2_ (−2.11 ppm) 283.1279 C_13_ H_19_ O_5_ N_2_ (−3.13 ppm) 257.1152 C_11_ H_17_ O_5_ N_2_ (−2.9 ppm) 243.0974 C_10_ H_15_ O_5_ N_2_ (−3.4 ppm) 242.1260 C_11_ H_18_ O_4_ N_2_ (−2.67 ppm) 225.0865 C_10_ H_13_ O_4_ N_2_ (−4.63 ppm) 211.1078 C_10_ H_15_ O_3_ N_2_ (−1.08 ppm) 199.1077 C_9_ H_15_ O_3_ N_2_ (−0.54 ppm)	265.1186 C_13_ H_17_ O_4_ N_2_ (−1.52 ppm) 239.1482 C_12_ H_19_ O_3_ N_2_ (−0.63 ppm) 221.1377 C_12_ H_17_ O_2_ N_2_ (−0.29 ppm)	
B	319.1495	C_13_ H_23_ O_7_ N_2_	304.1265 C_12_ H_20_ O_7_ N_2_ (1.88 ppm) 301.1392 C_13_ H_21_ O_6_ N_2_ (−2.46 ppm) 289.1394 C_12_ H_21_ O_6_ N_2_ (−2.46 ppm) 275.1237 C_11_ H_19_ O_6_ N_2_ (−2.15 ppm) 245.1132 C_10_ H_17_ O_5_ N_2_ (−2.60 ppm)	257.1237 C_11_ H_17_ O_5_ N_2_ (−2.40 ppm) 230.0897 C_9_ H_14_ O_5_ N_2_ (−3.94 ppm) 227.1023 C_10_ H_15_ O_4_ N_2_ (−1.02 ppm) 209.0917 C_10_ H_13_ O_3_ N_2_ (−0.98 ppm) 199.1076 C_9_ H_15_ O_3_ N_2_ (−0.95 ppm) 197.0919 C_9_ H_13_ O_3_ N_2_ (−0.95 ppm) 186.0996 C_8_ H_14_ O_3_ N_2_ (−0.89 ppm)	
C	331.1499	C_14_ H_23_ O_7_ N_2_	316.1264 C_13_ H_20_ O_7_ N_2_ (−1.96 ppm) 313.1393 C_14_ H_21_ O_6_ N_2_ (−0.71 ppm) 301.1035 C_12_ H_17_ O_7_ N_2_ (−0.09 ppm) 295.1289 C_14_ H_19_ O_5_ N_2_ (−0.74 ppm) 272.1367 C_12_ H_20_ O_5_ N_2_ (−2.17 ppm) 241.1182 C_11_ H_17_ O_4_ N_2_ (−0.64 ppm) 228.1468 C_11_ H_20_ O_3_ N_2_ (−2.64 ppm) 213.1234 C_10_ H_17_ O_3_ N_2_ (−1.73 ppm) 197.1286 C_10_ H_17_ O_2_ N_2_ (−0.4 ppm)		
D	361.1601	C_15_ H_25_ O_8_ N_2_	346.1361 C_14_ H_22_ O_8_ N_2_ (−4.35 ppm) 317.1346 C_13_ H_21_ O_7_ N_2_ (−0.27 ppm) 315.1192 C_13_ H_19_ O_7_ N_2_ (0.01 ppm) 302.1472 C_13_ H_22_ O_6_ N_2_ (−1.97 ppm) 271.1288 C_12_ H_19_ O_5_ N_2_ (−0.54 ppm) 258.1574 C_12_ H_22_ O_4_ N_2_ (−2.04 ppm) 227.1388 C_11_ H_19_ O_3_ N_2_ (−3.72 ppm)		
E	363.1398	C_14_ H_23_ O_9_ N_2_	348.1166 C_13_ H_20_ O_9_ N_2_ (−0.8 ppm) 345.1292 C_14_ H_21_ O_8_ N_2_ (−1.68 ppm) 330.1161 C_13_ H_18_ O_8_ N_2_ (−0.62 ppm) 319.1134 C_12_ H_19_ O_8_ N_2_ (−2.04 ppm) 304.1263 C_12_ H_20_ O_7_ N_2_ (−2.6 ppm) 286.1160 C_12_ H_18_ O_6_ N_2_ (−1.8 ppm) 283.0923 C_12_ H_15_ O_6_ N_2_ (−2.9 ppm) 239.1022 C_11_ H_15_ O_4_ N_2_ (−4.27 ppm)	309.1081 C_14_ H_17_ O_6_ N_2_ (−1.39 ppm) 301.1029 C_12_ H_17_ O_7_ N_2_ (−0.76 ppm)	
F	367.1169	C_13_ H_23_ O_8_ N_2_ S	352.0928 C_12_ H_20_ O_8_ N_2_ S (-2.38 ppm) 349.1851 C_13_ H_21_ O_7_ N_2_ S 323.1699 C_12_ H_23_ O_6_ N_2_ S 321.0742 C_11_ H_17_ O_7_ N_2_ S 308.0671 C_10_ H_16_ O_7_ N_2_ S (−2.5 ppm)	334.0822 C_12_ H_18_ O_7_ N_2_ S 331.1648 C_13_ H_19_ O_6_ N_2_ S 305.1586 C_11_ H_17_ O_6_ N_2_ S 303.1796 C_11_ H_15_ O_6_ N_2_ S 287.1890 C_12_ H_19_ O_4_ N_2_ S	290.093 C_11_ H_18_ O_5_ N_2_ S (-2.7 ppm) 277.0484 C_9_ H_13_ O_6_ N_2_ S 228.1101 C_11_ H_20_ O_3_ N_2_
G	411.1063	C_14_ H_23_ O_10_ N_2_ S	396.083 C_13_ H_20_ O_10_ N_2_ S (-1.48 ppm) 393.096 C_14_ H_21_ O_9_ N_2_ S (−1.64 ppm) 378.072 C_13_ H_18_ O_9_ N_2_ S 360.0623 C_13_ H_16_ O_8_ N_2_ S 352.0931 C_12_ H_20_ O_8_ N_2_ S 334.082 C_12_ H_18_ O_7_ N_2_ S 308.067 C_10_ H_16_ O_7_ N_2_ S 228.1104 C_10_ H_16_ O_4_ N_2_ 210.0996 C_10_ H_14_ O_3_ N_2_		

**Table 6:** Proposed structures of seven candidate-MAAs detected in the model algae *Gymnogongrus devoniensis*. The following structures were selected with the greatest FISh scores by comparing experimental and in silico fragmentation.

	Compound A	Compound B
Name	2-{[(3Z)-5-hydroxy-3-[(1-hydroxyethyl)imino]-5-(hydroxymethyl)-2-methoxycyclohex-1-en-1-yl]amino}prop-2-enoic acid	3-hydroxy-2-({(3E)-5-hydroxy-5-(hydroxymethyl)-3-[(hydroxymethyl)imino]-2-methoxycyclohex-1-en-1-yl}amino)butanoic acid
Structure	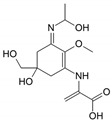	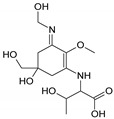
Monoisotopic mass (Da)	300.1321	318.1425
FISh score (%)	62	53
	Compound C	Compound D
Name	2-[(E)-{3-[(1-carboxyethyl)amino]-5-hydroxy-5-(hydroxymethyl)-2-methoxycyclohex-2-en-1-ylidene}amino]propanoic acid	2-{[(3E)-3-[(1-carboxyethyl)imino]-5-hydroxy-5-(hydroxymethyl)-2-methoxycyclohex-1-en-1-yl]amino}-3-hydroxybutanoic acid
Structure	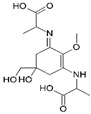	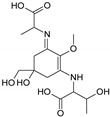
Monoisotopic mass (Da)	330.1420	360.1522
FISh score (%)	59	54
	Compound E	Compound F
Name	2-{(E)-[3-{[carboxy(hydroxy)methyl]amino}-5-hydroxy-5-(hydroxymethyl)-2-methoxycyclohex-2-en-1-ylidene]amino}-3-hydroxybutanoic acid	{[(3E)-5-(hydroxymethyl)-3-[(2-hydroxypropyl)imino]-2-methoxy-5-(sulfinooxy)cyclohex-1-en-1-yl]amino}acetic acid
Structure	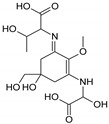	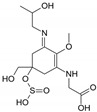
Monoisotopic mass (Da)	362.1320	366.1096
FISh score (%)	52	60
	Compound G	
Name	2-[(E)-{3-[(carboxymethyl)amino]-5-hydroxy-2-methoxy-5-[(sulfinooxy)methyl]cyclohex-2-en-1-ylidene}amino]-3-hydroxybutanoic acid	
Structure	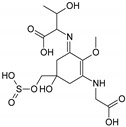	
Monoisotopic mass (Da)	410.0995	
FISh score (%)	54	

**Table 7:** Semi-quantitative analysis of MAAs detected in *Gymnogongrus devoniensis*. Amounts of MAA was estimated with an error of 10% regarding the signal intensities observed between the purified MAAs porphyra-334, shinorine and palythine injected at the same concentrations.

MAAs	Monoisotopic Mass (Da)	Area Max (10^5^)	Amounts (µg.g^−1^ DW)	Error (µg.g^−1^ DW)
Porphyra-334	346.1372	693	2100	0
Shinorine	332.1214	85.2	430	0
Palythine	244.1051	122	1530	0
Compound E	362.1320	1.3	4	0.40
Aplysiapalythine-B	272.1367	76.5	243	24.3
Palythinol	302.1475	11.3	35.8	3.6
Mycosporine-2-glycine	302.1112	3.6	11.3	1.1
Mycosporine methyl-amine threonine	302.1475	2.4	7.7	0.8
Asterina-330	288.1316	187	593	59.3
Palythene/Usujirene	284.1368	137	434	43.4
Compound B	318.1425	1.6	5.2	0.5
Compound D	360.1522	0.28	0.9	0.1
Mycosporine-glycine-valine	344.1579	1.2	3.8	0.4
Compound C	330.1420	1	3.4	0.3
Palythine glutamic-acid	316.1266	6.3	19.8	2
Compound F	366.1092	0.5	1.5	0.2
Palythine-serine	274.1158	0.5	1.5	0.2
Compound G	410.0990	0.5	1.6	0.2
Compound A	300.1320	2.7	8.4	0.8
Aplysiapalythine-A	302.1475	2	6.6	0.7
Mycosporine-glycine	245.0899	0.9	2.9	0.3

**Table 8:** Candidate-MAAs detected in four different algal species using the Fragment Ion Search (FISh) in ddMS^2^/MS3 Untargeted Analysis and validated by subsequent Targeted Analysis.

MAAs	Formula	MW (Da)	[M+H]^+^ (*m*/*z*)	Retention Time (min)	Number of Fragment Ions (/8)			
					*Porphyra rosengurtii*	*Gelidium sesquipedale*	*Halopithys incurva*	*Cystoseira tamariscifolia*
**Palythine**	**C_10_ H_16_ O_5_ N_2_**	**244.1051**	**245.1124**	**11.16**	8	8	6	7
Unknown	C_12_ H_24_ O_3_ N_2_	244.1786	245.1858	11.30	8			
**Mycosporine-glycine**	**C_10_ H_15_ O_6_ N**	**245.0891**	**246.0963**	**8.56**			5	
Unknown	C_10_ H_17_ O_6_ N	247.1051	248.1124	11.04		8		
**Aplysiapalythine-B**	**C_12_ H_20_ O_5_ N_2_**	**272.1364**	**273.1437**	**9.01**		5		
**Palythine-serine**	**C_11_ H_18_ O_6_ N_2_**	**274.1157**	**275.1230**	**11.54**	8	5		
**Palythene/Usujirene**	**C_13_ H_20_ O_5_ N_2_**	**284.1369**	**285.1438**	**7.82**			8	
Unknown	C_12_ H_18_ O_6_ N_2_	286.1163	287.1236	10.54	7			
**Asterina-330**	**C_12_ H_20_ O_6_ N_2_**	**288.1315**	**289.1387**	**11.05**	7	8	7	
**Palythine-threonine**	**C_12_ H_20_ O_6_ N_2_**	**288.1321**	**289.1394**	**10.48**	7			
**Palythinol**	**C_13_ H_22_ O_6_ N_2_**	**302.1471**	**303.1544**	**10.20**			6	
**Palythine glutamic-acid**	**C_13_ H_20_ O_7_ N_2_**	**316.1266**	**317.1341**	**9.87**	7			
**Compound B**	C_13_ H_22_ O_7_ N_2_	318.1424	319.1492	10.24	6			
**Shinorine**	**C_13_ H_20_ O_8_ N_2_**	**332.1214**	**333.1286**	**10.53**	8	8	5	7
**Porphyra-334**	**C_14_ H_22_ O_8_ N_2_**	**346.1371**	**347.1447**	**10.16**	7		7	6
Unknown	C_14_ H_22_ O_8_ N_2_	346.1370	347.1437	9.48			5	
**Compound F**	C_13_ H_22_ O_8_ N_2_ S	366.1088	367.1161	11.03		5		
**Compound G**	C_14_ H_22_ O_10_ N_2_ S	410.0991	411.1064	10.53	8			

MAAs in bold letters correspond to those found in prior Targeted Analysis. MAAs identified in prior analysis of the model algal extract *Gymnogongrus devoniensis* were emphasized in bold letters.

## Supplementary Materials

The following are available online at https://www.mdpi.com/2076-3921/9/12/1185/s1, Figure S1. (a) MS method developed for targeted screening approach of MAAs. (b) Flowchart of the Targeted Screening Approach of MAAs using an Orbitrap MS based on the Top3 MS^2^ analysis. Figure S2 (a,b,c,d). XIC of MAAs (3ppm) in *Porphyra rosengurtii, Gelidium sesquipedale, Halopithys incurva* and *Cystoseira tamarsiscifolia* separated on the Zic-cHilic column. Figure S3. Fragmentation pathways of palythine (a) and porphyra-334 (b) obtained from their CID30 MS^2^ spectrum. Figure S4. CID30 MS^2^ spectrum of *m*/*z* 301.1392. Characteristic neutral and radical losses were annotated between the fragment ions affected on the spectrum. Figure S5. MS^2^ spectum of asterina-330 (*m*/*z* 289.1394) obtained in targeted screening analysis with a stepped collision energy at HCD50 +/− 20. Figure S6 (a–f). Structural elucidation of six candidate-MAAs using ddMS^2^ spectra obtained in untargeted analysis and their multistage fragmentation after fraction collection. Table S1. Multiple structural predictions of three candidate-MAAs among the set of seven candidate-MAAs elucidated.
