# Supplementary material for: Untargeted Analysis for Mycosporines and Mycosporine-Like Amino Acids by Hydrophilic Interaction Liquid Chromatography (HILIC)—Electrospray Orbitrap MS2/MS3
Source: Antioxidants (Basel). 2020 Nov 26;9(12):1185. doi: 10.3390/antiox9121185 (PMC7760333; doi:10.3390/antiox9121185)
Supplement: Supplementary file 1 [file antioxidants-09-01185-s001.pdf]

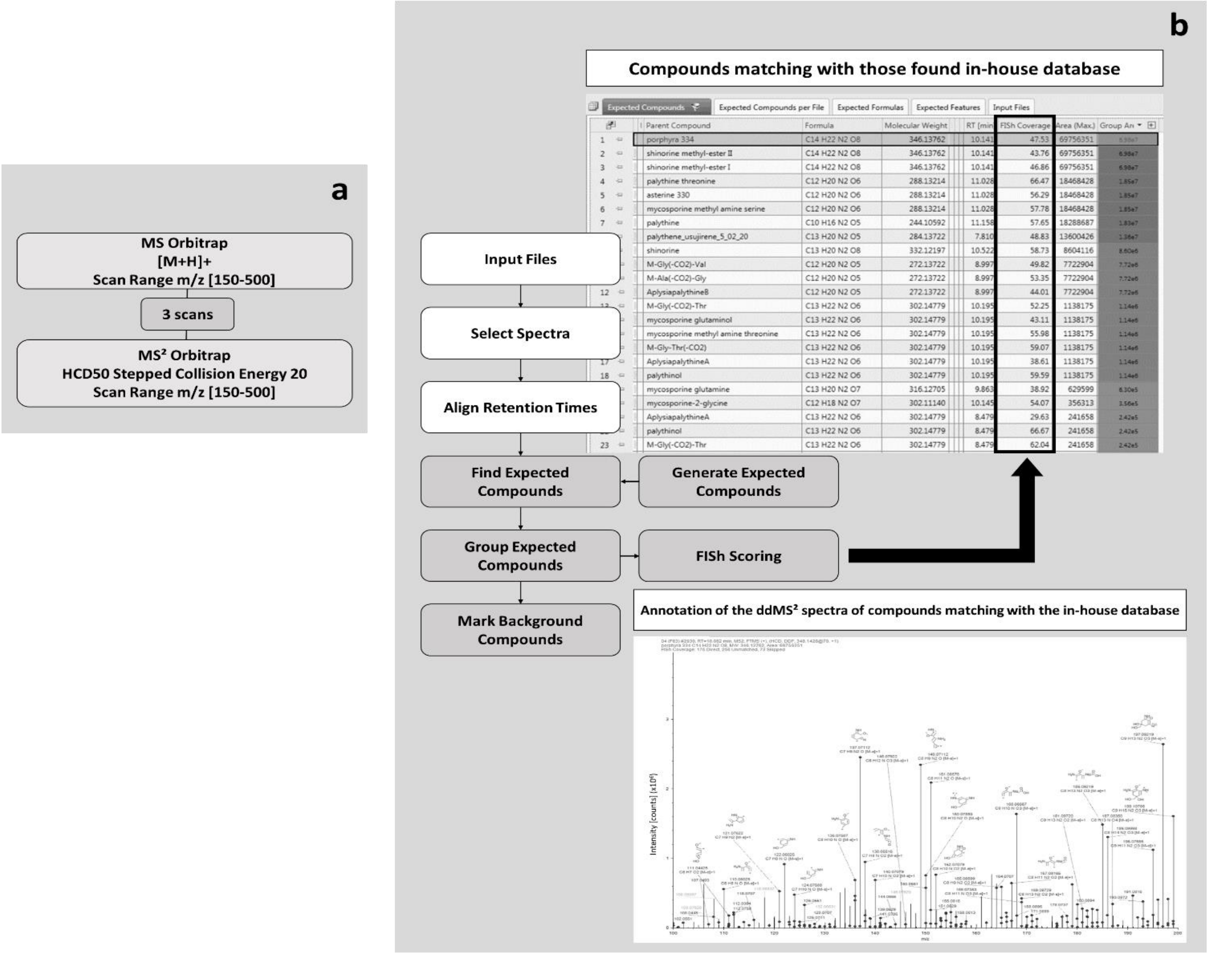

Figure 1. (**a**) MS method developed for targeted screening approach of MAAs. A Top3 MS² analysis was carried out to make the inventory of MAAs in the model algal extract *Gymnogongrus devoniensis*. Full MS Orbitrap (OT) settings: resolution 140000, mass range 150-500 m/z, dynamic exclusion 5 sec and intensity threshold 2e4. MS² scans settings: HCD50 resolution 60000, stepped collision energy 20, isolation width 2 m/z. For every detected mass, a FISh score was calculated to estimate the percentage of fragment ions generated in-silico matching with the collected spectral data. (**b**) Flowchart of the Targeted Screening Approach of MAAs using an Orbitrap MS based on the Top3 MS² analysis. Targeted workflow designed on Compound Discoverer™ to detect MAAs in the model algae matching with those listed in an in-house database. Description of the role of the following nodes: Find Expected Compounds. Research of compounds in the compound ion list provided by one or more Generate Exepected Compounds nodes. Generate Expected Compounds. Generation of a list of m/z values for the expected compounds to find in the sample. The list includes the parent compound and its possible transformation products. Group Expected Compounds. Combination of chromatographic peaks on the basis of their chemical formula and retention times. FISh Scoring. Calculation of a score for compounds detected by the Find Expected Compounds node and annotation of the fragmentation spectra for these compounds.

(a)

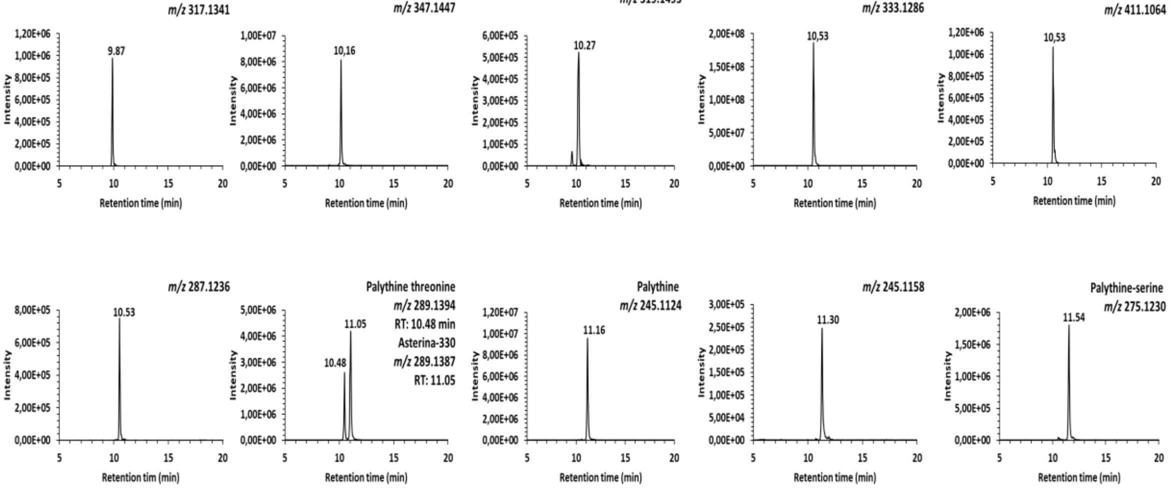
Palythine-glutamic acid
m/z 317.1341
9.87
Porphyra-334
m/z 347.1447
10,16
m/z 319.1493
10.27
Shinorine
m/z 333.1286
10,53
Compound G
m/z 411.1064
10,53
m/z 287.1236
10.53
Palythine threonine
m/z 289.1394
RT: 10.48 min
Asterina-330
m/z 289.1387
RT: 11.05
10.48
11.05
Palythine
m/z 245.1124
11.16
m/z 245.1158
11.30
Palythine-serine
m/z 275.1230
11.54
Intensity
Retention time (min)
Retention tim (min)

(b)

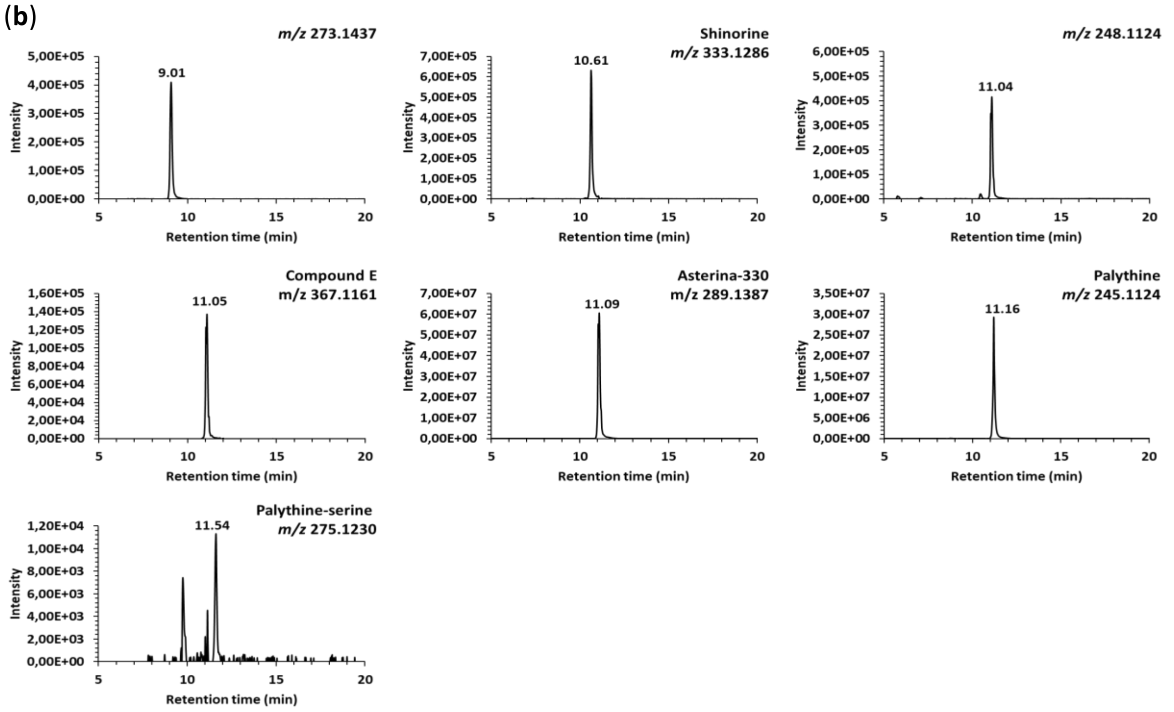
m/z 273.1437
9.01
Shinorine
m/z 333.1286
10.61
m/z 248.1124
11.04
Compound E
m/z 367.1161
11.05
Asterina-330
m/z 289.1387
11.09
Palythine
m/z 245.1124
11.16
Palythine-serine
m/z 275.1230
11.54
Intensity
Retention time (min)

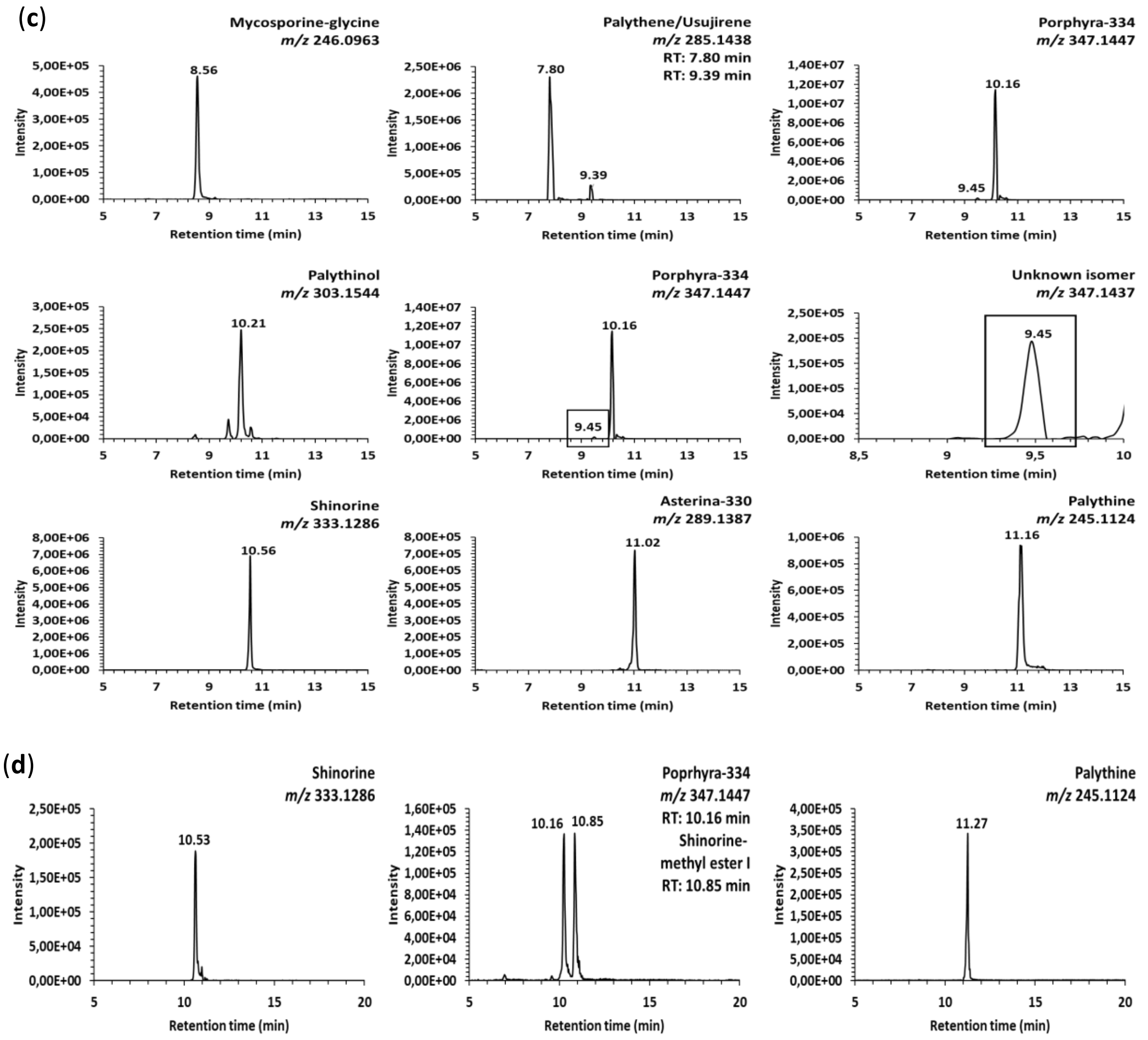

Figure 2. XIC of MAAs (3ppm) in *Porphyra rosengurtii* (**a**), *Gelidium sesquipedale* (**b**), *Halopithys incurva* (**c**) and *Cystoseira tamarsiscifolia* (**d**) separated on the Zic-cHilic column. Note that in *Cystoseira tamariscifolia* extract, the XIC of m/z 347.1447 displays two peaks among which the Porphyra-334 (RT : 10.16 min). The latter could correspond to the shinorine-methyl ester (RT : 10.85 min) with a FISh coverage of 73% calculated in targeted screening analysis. However, as the number of characteristic fragment ions was estimated at three, the MAA was not reported in Table 8, section 4.

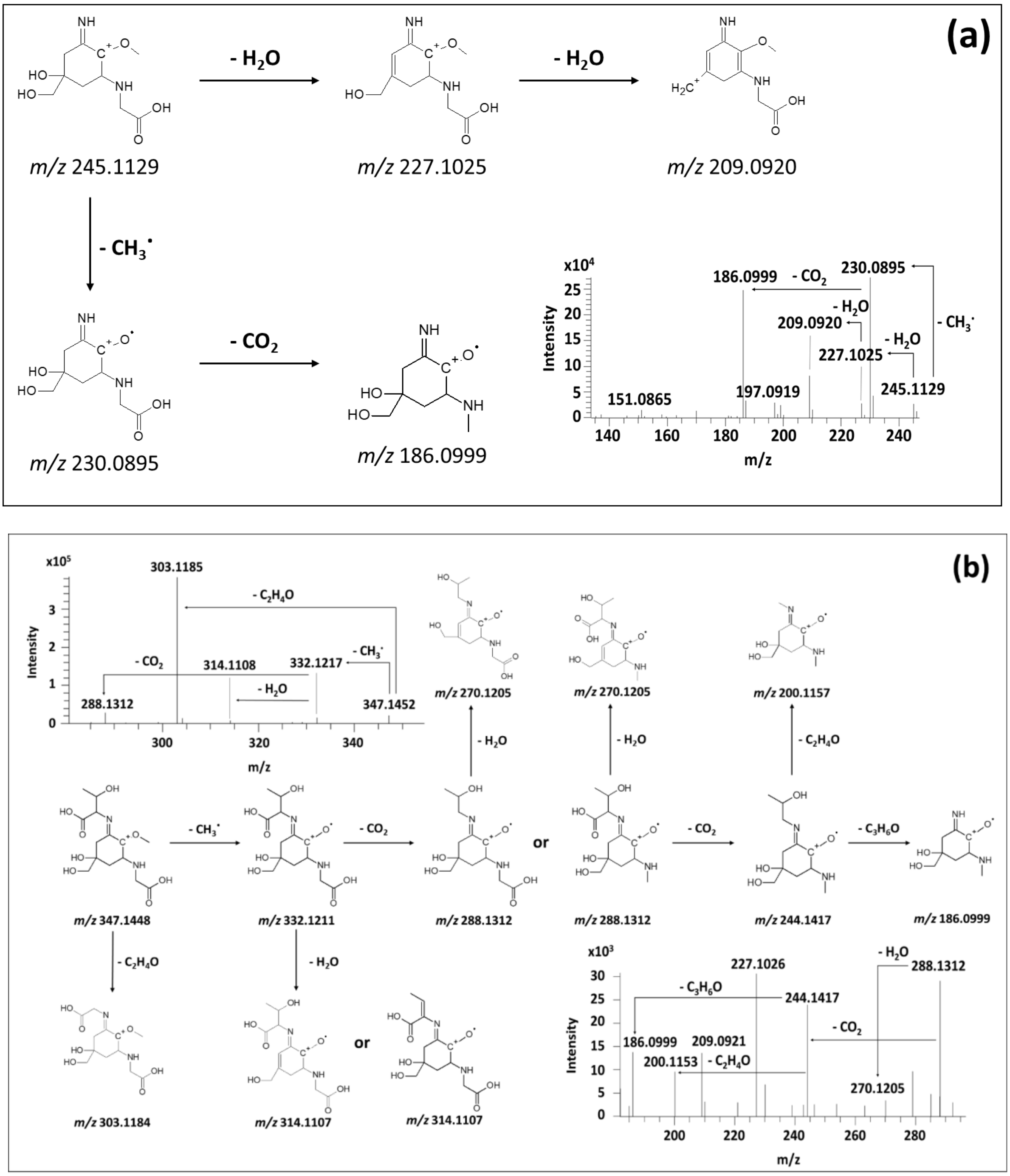

Figure 3. Fragmentation pathways of palythine (**a**) and porphyra-334 (**b**) obtained from their CID30 MS^2 spectrum.

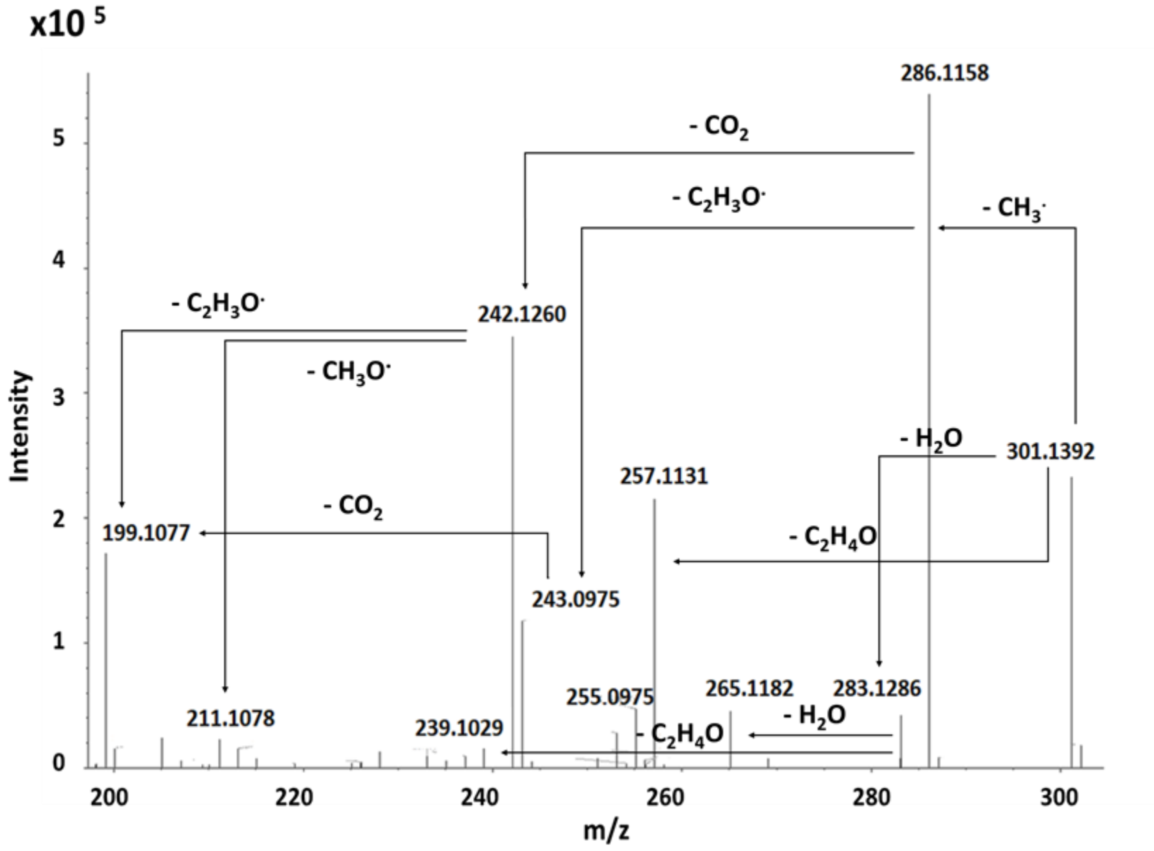

Figure 4. CID30 MS² spectrum of m/z 301.1392. Characteristic neutral and radical losses were annotated between the fragment ions affected on the spectrum.

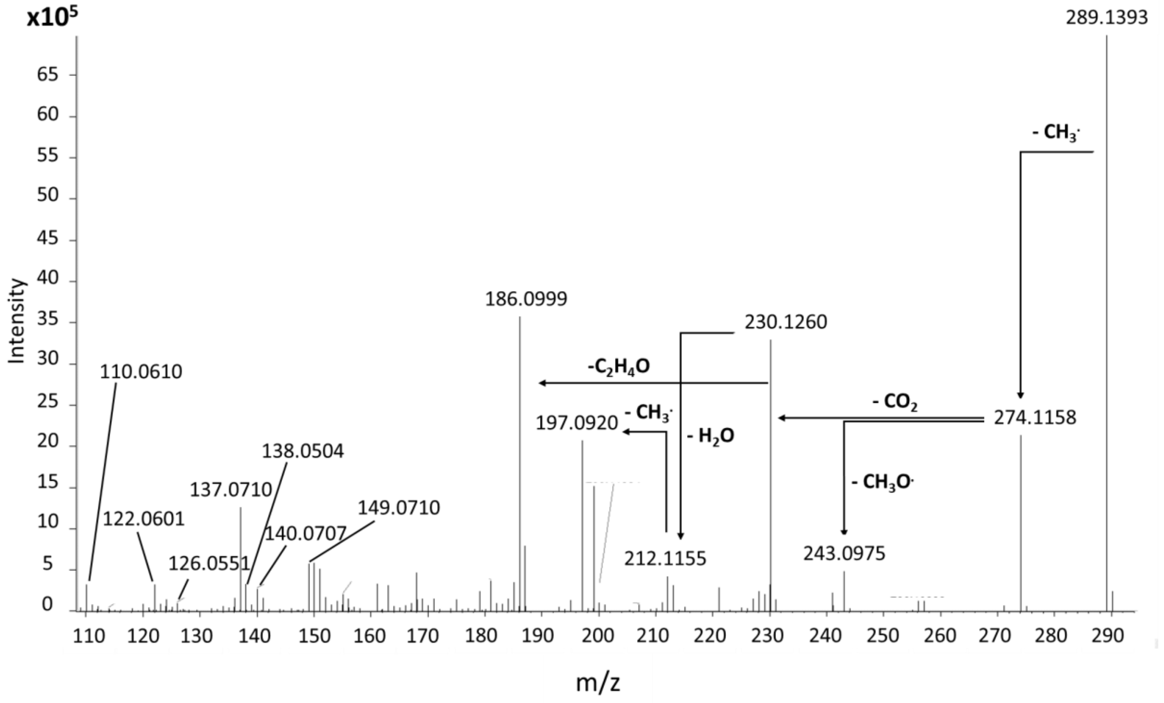

Figure 5. MS² spectum of asterina-330 (m/z 289.1394) obtained in targeted screening analysis with a stepped collision energy at HCD50 +/- 20.

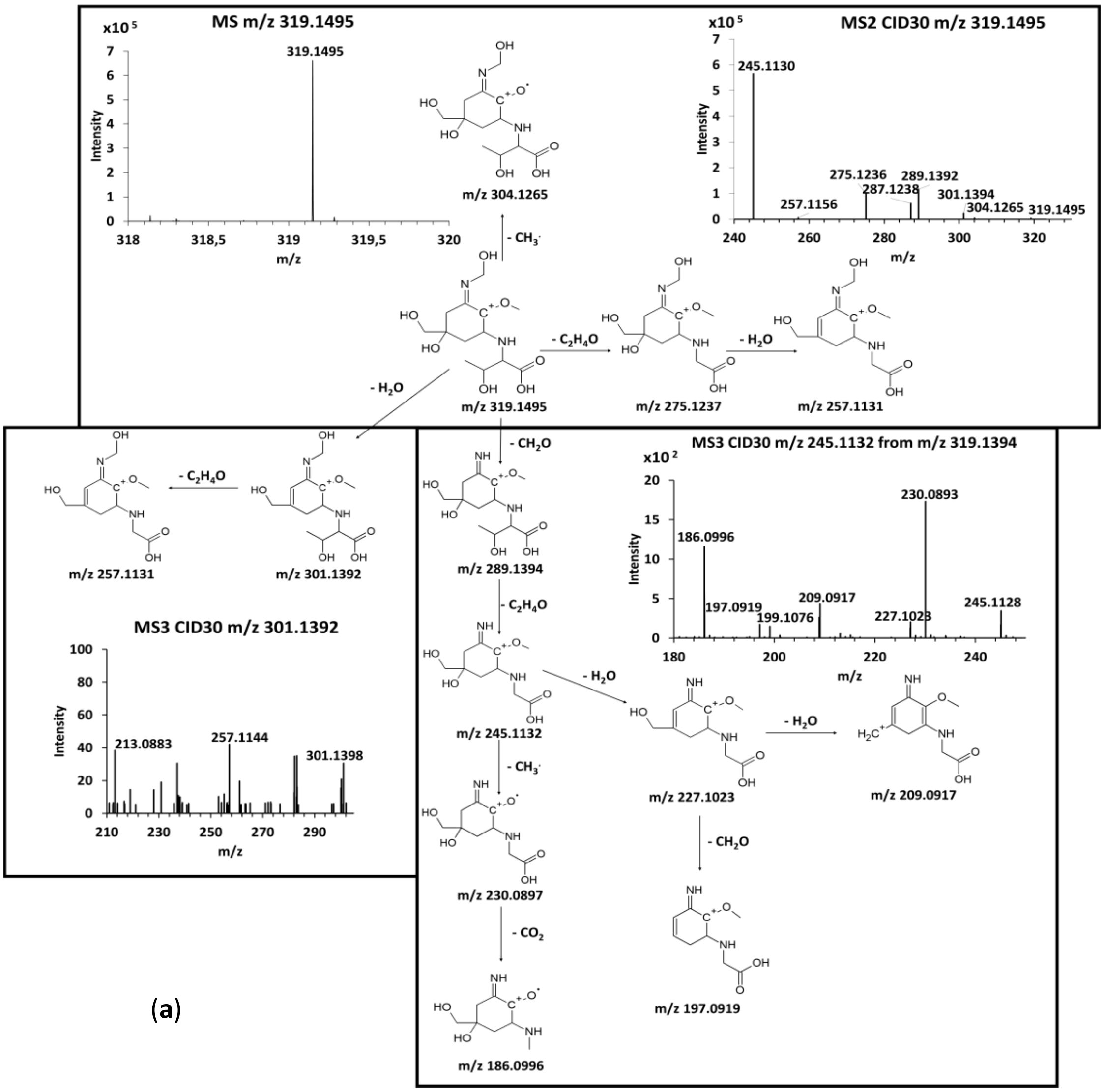
MS m/z 319.1495
x10 5
319.1495
Intensity
m/z
MS2 CID30 m/z 319.1495
245.1130
275.1236
289.1392
287.1238
301.1394
257.1156
304.1265
319.1495
m/z 304.1265
- CH3·
m/z 319.1495
- C2H4O
m/z 275.1237
- H2O
m/z 257.1131
m/z 301.1392
MS3 CID30 m/z 301.1392
213.0883
257.1144
301.1398
- CH2O
m/z 289.1394
m/z 245.1132
m/z 230.0897
- CO2
m/z 186.0996
MS3 CID30 m/z 245.1132 from m/z 319.1394
x10 2
230.0893
186.0996
197.0919
209.0917
199.1076
227.1023
245.1128
m/z 227.1023
m/z 209.0917
m/z 197.0919

(a)

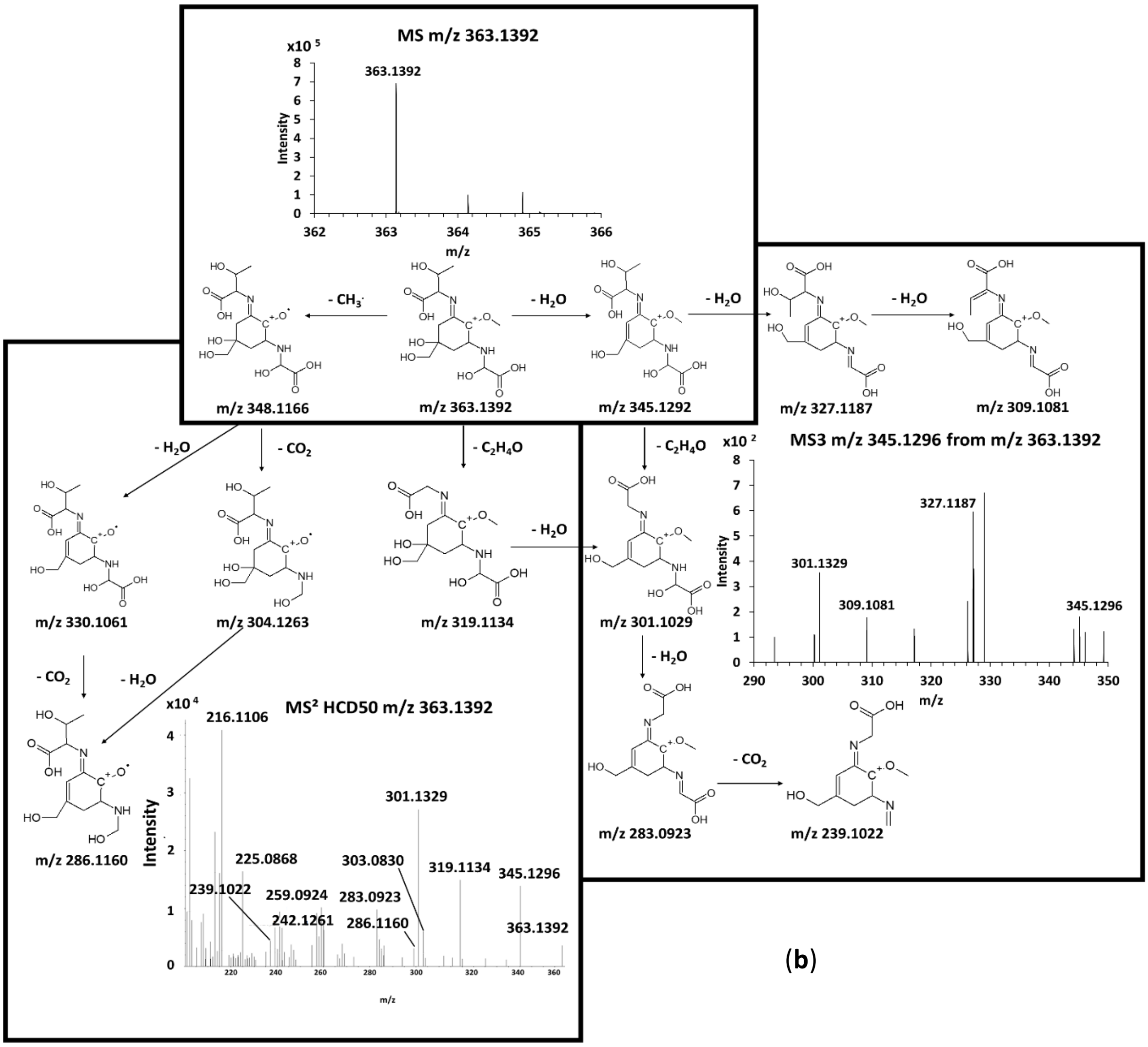
MS m/z 363.1392
363.1392
Intensity
m/z
- CH3·
- H2O
- H2O
- H2O
m/z 348.1166
m/z 363.1392
m/z 345.1292
m/z 327.1187
m/z 309.1081
- H2O
- CO2
- C2H4O
- C2H4O
MS3 m/z 345.1296 from m/z 363.1392
327.1187
301.1329
309.1081
345.1296
m/z 330.1061
m/z 304.1263
m/z 319.1134
m/z 301.1029
- H2O
- CO2
- H2O
MS² HCD50 m/z 363.1392
216.1106
301.1329
225.0868
303.0830
319.1134
345.1296
239.1022
259.0924
283.0923
242.1261
286.1160
363.1392
m/z 286.1160
m/z 283.0923
- CO2
m/z 239.1022

(**b**)

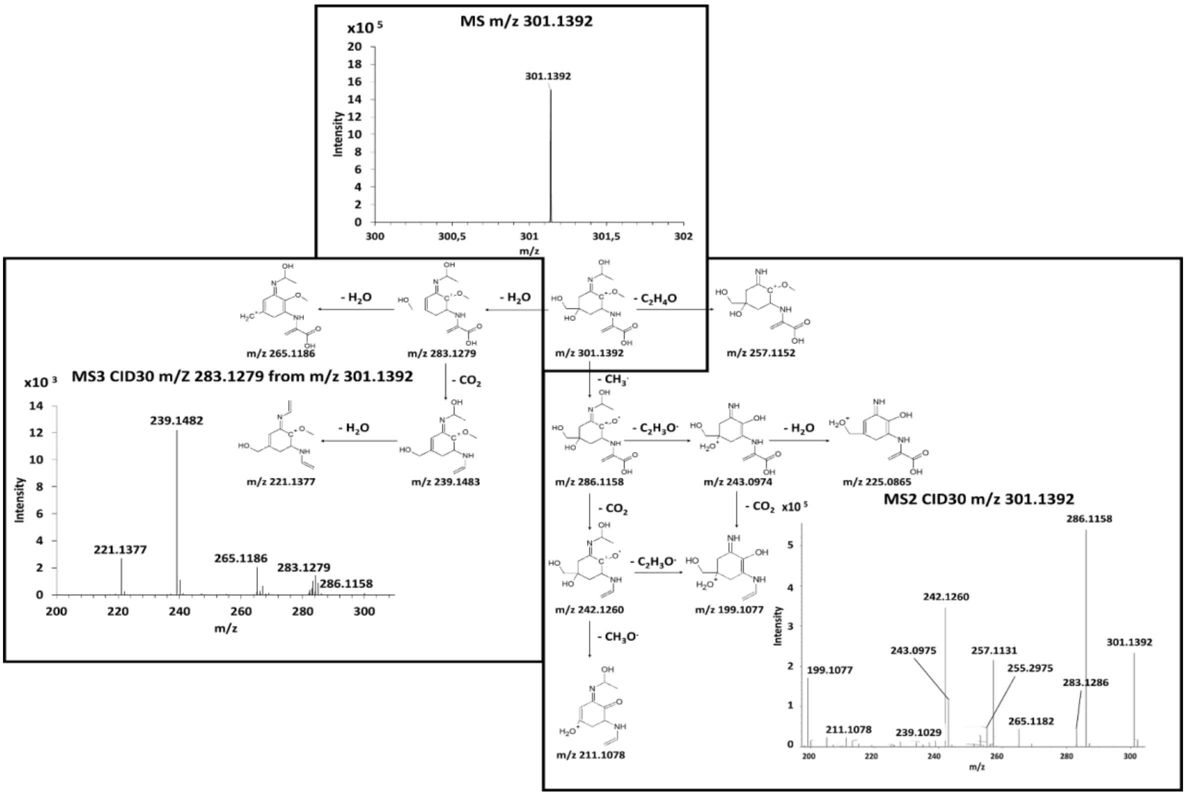

MS m/z 301.1392
x10 5
Intensity
301.1392
m/z
- H2O
- H2O
- C2H4O
m/z 265.1186
m/z 283.1279
m/z 301.1392
m/z 257.1152
MS3 CID30 m/Z 283.1279 from m/z 301.1392
x10 3
- CO2
- CH3·
- H2O
- C2H3O·
- H2O
m/z 221.1377
m/z 239.1483
m/z 286.1158
m/z 243.0974
m/z 225.0865
239.1482
221.1377
265.1186
283.1279
286.1158
- CO2
- CO2
- C2H3O·
m/z 242.1260
m/z 199.1077
- CH3O·
m/z 211.1078
MS2 CID30 m/z 301.1392
x10 5
286.1158
242.1260
243.0975
257.1131
255.2975
301.1392
199.1077
283.1286
211.1078
265.1182
239.1029
m/z

(**d**)

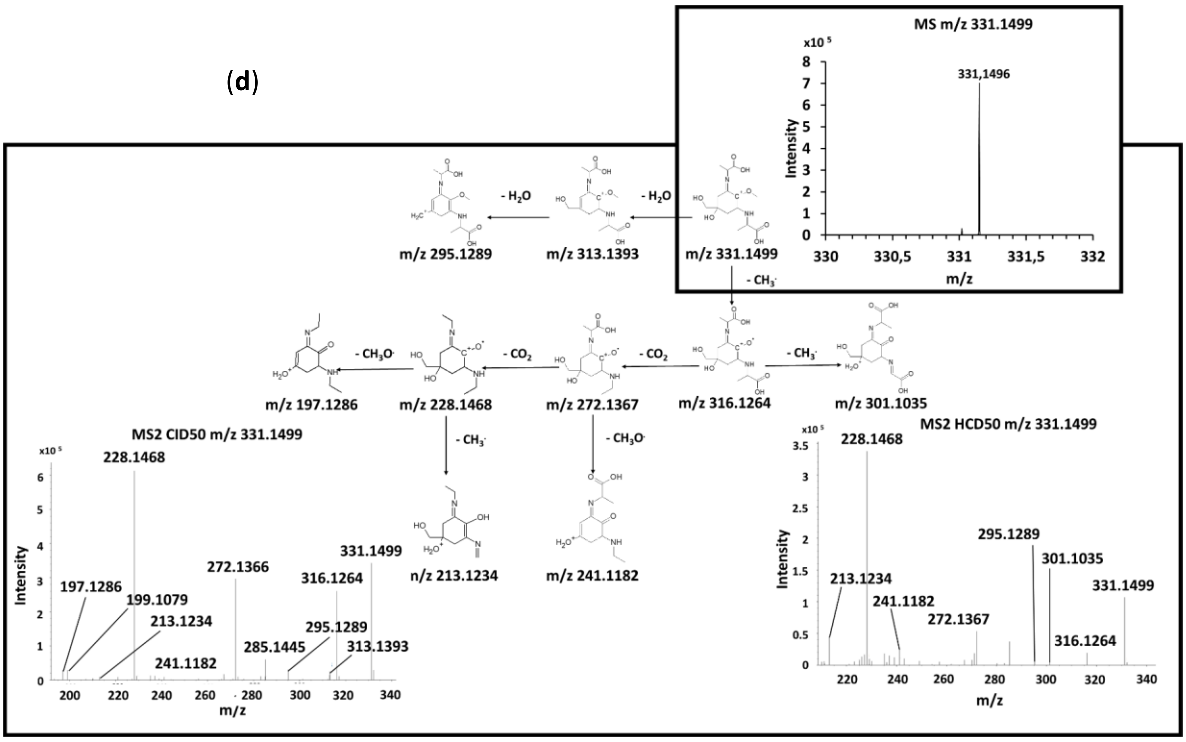

MS m/z 331.1499
x10 5
331,1496
Intensity
m/z
- H2O
- H2O
m/z 295.1289
m/z 313.1393
m/z 331.1499
- CH3·
- CH3O·
- CO2
- CO2
- CH3·
m/z 197.1286
m/z 228.1468
m/z 272.1367
m/z 316.1264
m/z 301.1035
MS2 CID50 m/z 331.1499
MS2 HCD50 m/z 331.1499
- CH3·
- CH3O·
n/z 213.1234
m/z 241.1182
228.1468
331.1499
272.1366
316.1264
197.1286
199.1079
213.1234
241.1182
285.1445
295.1289
313.1393
295.1289
301.1035
213.1234
241.1182
272.1367
316.1264

(e)

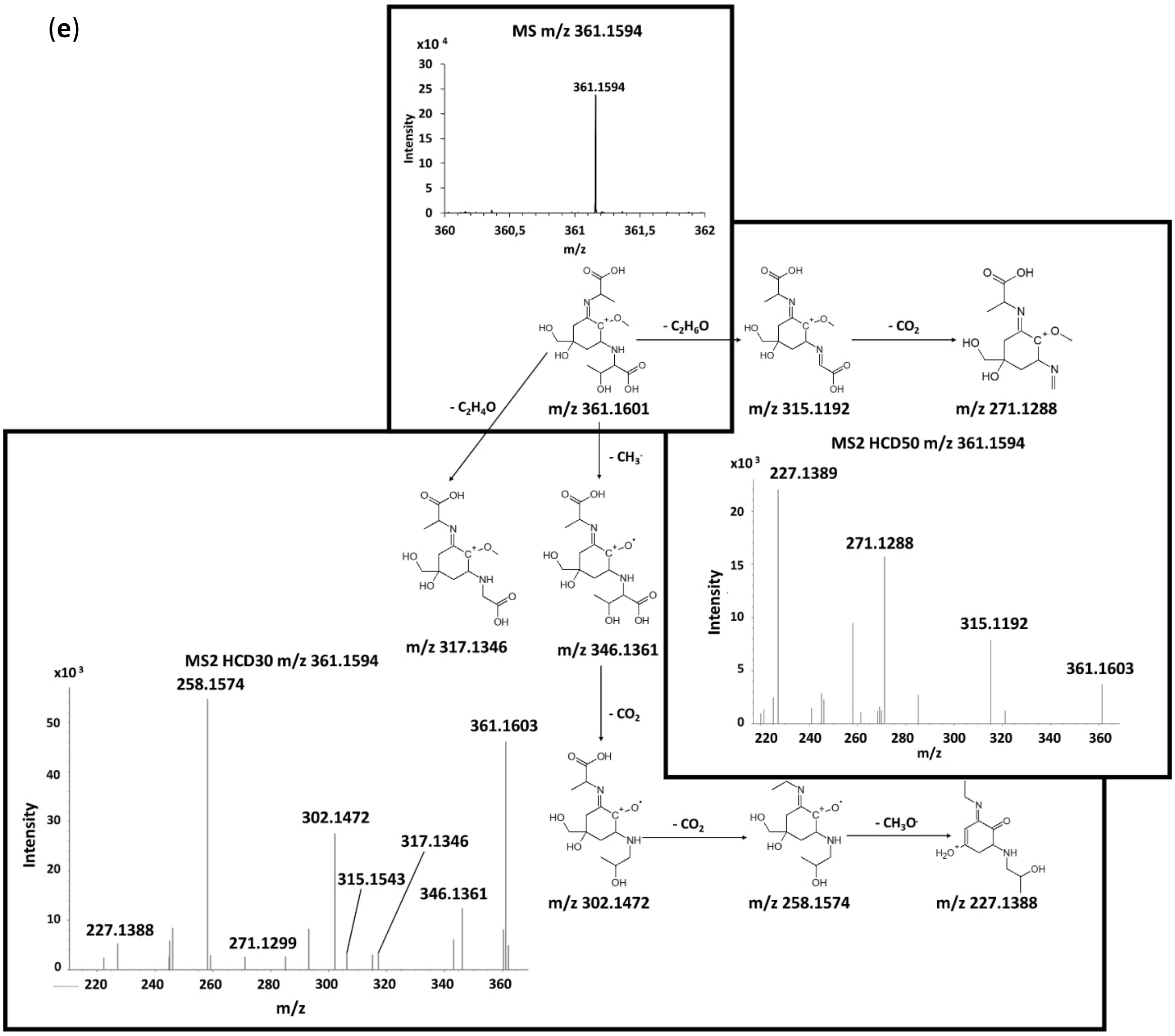

MS m/z 361.1594
x10 4
361.1594
Intensity
m/z
- C$_2$H$_6$O
- CO$_2$
- C$_2$H$_4$O
- CH$_3$
m/z 361.1601
m/z 315.1192
m/z 271.1288
MS2 HCD50 m/z 361.1594
x10 3
227.1389
271.1288
315.1192
361.1603
m/z 317.1346
m/z 346.1361
MS2 HCD30 m/z 361.1594
258.1574
361.1603
302.1472
317.1346
315.1543
346.1361
227.1388
271.1299
- CO$_2$
- CH$_3$O
m/z 302.1472
m/z 258.1574
m/z 227.1388

(f)

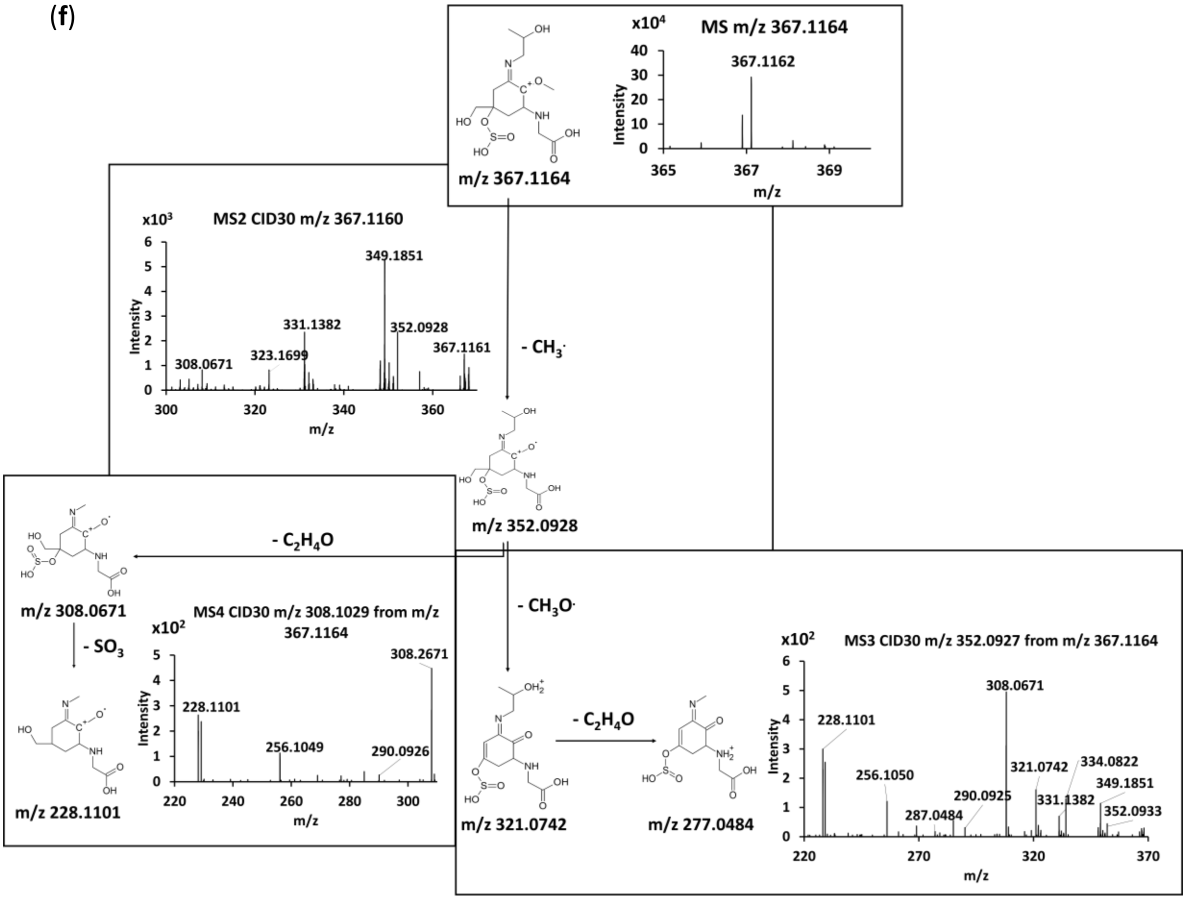

Figure 6. (a–f). Structural elucidation of six candidate-MAAs using ddMS[2] spectra obtained in untargeted analysis and their multistage fragmentation after fraction collection.

Table 1. Multiple structural predictions of three candidate-MAAs among the set of seven candidate-MAAs elucidated.

Compound A			
2-{[(3Z)-5-hydroxy-3-[(1-hydroxyethyl)imino]-5-(hydroxymethyl)-2-methoxycyclohex-1-en-1-yl]amino}prop-2-enoic acid	2-{[(3Z)-5-hydroxy-3-[(2-hydroxyethyl)imino]-5-(hydroxymethyl)-2-methoxycyclohex-1-en-1-yl]amino}prop-2-enoic acid	2-{[(3Z)-5-hydroxy-3-{[(E)-2-hydroxyethenyl]imino}-5-(hydroxymethyl)-2-methoxycyclohex-1-en-1-yl]amino}propanoic acid	(3E)-4-hydroxy-2-{[(3Z)-5-hydroxy-5-(hydroxymethyl)-2-methoxy-3-(methylimino)cyclohex-1-en-1-yl]amino}but-3-enoic acid
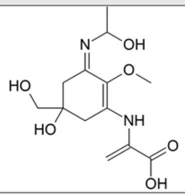	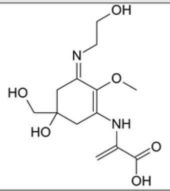	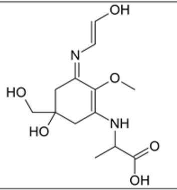	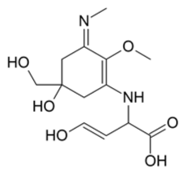
FISh Score: 62%	FISh Score: 60%	FISh Score: 57%	FISh Score: 60%
Monoisotopic mass: 300.1321 Da<br>[M+H]+ m/z: 301.1392			

Compound C		
2-[(E)-{3-[(1-carboxyethyl)amino]-5-hydroxy-5-(hydroxymethyl)-2-methoxycyclohex-2-en-1-ylidene}amino]propanoic acid	2-{[(3Z)-3-[(2-carboxyethyl)imino]-5-hydroxy-5-(hydroxymethyl)-2-methoxycyclohex-1-en-1-yl]amino}propanoic acid	3-[(Z)-{3-[(2-carboxyethyl)amino]-5-hydroxy-5-(hydroxymethyl)-2-methoxycyclohex-2-en-1-ylidene}amino]propanoic acid
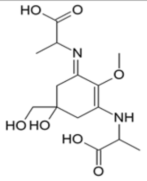	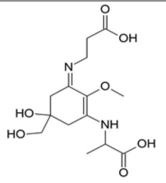	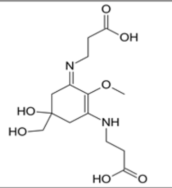
FISh Score: 59%	FISh Score: 57%	FISh Score: 58%
Monoisotopic mass: 330.1427 Da<br>[M+H]+ m/z: 331.1499		

Compound F		
{[(3E)-5-(hydroxymethyl)-3-[(2-hydroxypropyl)imino]-2-methoxy-5-(sulfinooxy)cyclohex-1-en-1-yl]amino}acetic acid	2-{[(3Z)-3-[(2-hydroxyethyl)imino]-5-(hydroxymethyl)-2-methoxy-5-(sulfinooxy)cyclohex-1-en-1-yl]amino}propanoic acid	3-hydroxy-2-{[(3Z)-5-(hydroxymethyl)-2-methoxy-3-(methylimino)-5-(sulfinooxy)cyclohex-1-en-1-yl]amino}butanoic acid
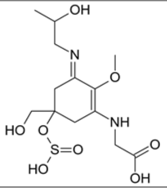	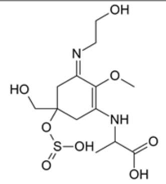	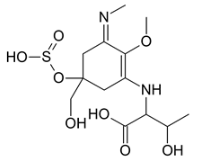
FISh Score: 60%	FISh Score: 59%	FISh Score: 50%
Monoisotopic mass: 366.1096 Da<br>[M+H]+ m/z: 367.1169		
